# First identification of ITM2B interactome in the human retina

**DOI:** 10.1038/s41598-021-96571-6

**Published:** 2021-08-26

**Authors:** J. Wohlschlegel, M. Argentini, C. Michiels, C. Letellier, V. Forster, C. Condroyer, Z. He, G. Thuret, C. Zeitz, T. Léger, I. Audo

**Affiliations:** 1 Sorbonne Université, INSERM, CNRS, Institut de la Vision, 17 rue Moreau, 75012 Paris, France; 2 Corneal Graft Biology, Engineering and Imaging Laboratory, Health Innovation Campus, Faculty of Medicine, Jean Monnet University, Saint-Etienne, France; 3 Department of Ophthalmology, University Hospital, Saint-Etienne, France; 4 Mass Spectrometry Laboratory, Institut Jacques Monod, UMR 7592, Université Paris Diderot, CNRS, Sorbonne Paris Cité, 75205 Paris, France; 5 Univ Rennes, Inserm, EHESP, Irset (Institut de Recherche en Santé, environnement et travail)-UMR_S 1085, 35000 Rennes, France; 6 CHNO des Quinze-Vingts, INSERM-DGOS CIC 1423, 28 rue de Charenton, 75012 Paris, France; 7 Department of Genetics, UCL-Institute of Ophthalmology, 11-43 Bath Street, London, EC1V 9EL UK

**Keywords:** Retina, Mass spectrometry, Neurophysiology

## Abstract

Integral Membrane Protein 2 B (ITM2B) is a type II ubiquitous transmembrane protein which role remains unclear. *ITM2B* mutations have been associated with different disorders: mutations leading to longer mutant proteins have been reported in two distinct Alzheimer-like autosomal dominant disorders with early-onset progressive dementia and cerebellar ataxia. Both disorders share neurological features including severe cerebral amyloid angiopathy, non-neuritic plaques, and fibrillary tangles as in Alzheimer disease. Our group reported a missense mutation in *ITM2B,* in an unusual retinal dystrophy with no dementia. This finding suggests a specific role of ITM2B in the retina. As the identification of retinal-specific ITM2B partners could bring new insights into the cellular functions of ITM2B, we performed quantitative proteomics of ITM2B interactome of the human retina. Overall, 457 ITM2B partners were identified with 8 of them involved in visual transduction. In addition, bulk Gene Ontology analyses showed that many ITM2B partners are involved in several other biological functions, such as microtubule organization, protein translation and interestingly, mitochondrial homeostasis. These data represent the first report of the ITM2B interactome in the human retina and may serve as a valuable inventory of new potential ITM2B partners for future investigations of ITM2B physiological functions and dysfunctions.

## Introduction

ITM2B (Integral membrane protein 2b also called BRI2, MIM#603904) is a single pass type II ubiquitous protein which function is still unclear. ITM2B is cleaved by different enzymes, furin or furin-like proteases, at its C-terminus, releasing a 23-amino acid peptide, called Bri23^1,2^. ITM2B is highly expressed in the brain and in the retina^3,4^. Mutations in this gene lead to different disorders in humans. *ITM2B* mutations leading to a longer protein product are responsible for two distinct autosomal dominant neurodegenerative disorders: the Familial British Dementia (FBD, MIM#176500)^5–7^ and the Familial Danish Dementia (FDD, MIM#117300)^8^. The diseases consist mainly of early-onset forms of progressive dementia with cerebellar ataxia and spasticity. FDD is also known as heredopathia ophthalmo-oto-encephalica^9^ with the first symptoms being the development of cataract and progressive deafness. Both FBD and FDD share neurological features including severe cerebral amyloid angiopathy, non-neuritic plaques^7^, and fibrillary tangles as in Alzheimer Disease (AD). The respective mutations lead to a longer protein at the C-terminus the cleavage of which releases, instead of Bri23, a 34-amino acid peptide named ADan in FDD and ABri in FBD. ADan^8^ and ABri^5^ are the major components of insoluble aggregates in the brain of affected subjects. More recently, another autosomal dominant mutation in *ITM2B* (c.782A>C, p.Glu261Ala) has been reported in an unusual retinal dystrophy associated with retinal ganglion cell abnormalities, inner retinal and cone dysfunctions. Furthermore, the phenotype is only restricted to the retina (MIM#616079)^3,10^, and none of the affected subjects present dementia, suggesting that ITM2B plays a specific role in the retina. Interestingly, the mutation identified in the retinal dystrophy^3^ is also located within the 23-amino acid C-terminal cleaved peptide, outlining the functional importance of this peptide.

In normal conditions, the furin or furin-like cleavage generates the mature form of ITM2B which is processed in the cis-Golgi apparatus. ITM2B may also be cleaved by ADAM10^11^ releasing an evolutionary conserved domain called the BRICHOS domain. Then, the remaining membrane-bound peptide is intramembranously proteolysed by Signal Peptidase-Like 2B (SPPL2B) releasing one intracellular domain in the cytosol and a small extracellular secreted peptide^12,13^.

In the brain, ITM2B interacts with AβPP (Amyloid β Precursor Protein) acting as an inhibitor of Amyloid β (Aβ) oligomerization^12–14^, a major component of amyloid plaques in AD and AD-like diseases. ITM2B has also been involved in neuronal functions and in particular in neurite outgrowth^15^. Recently, ITM2B has been shown to interact with APLP2 (APP-related protein 2), a protein belonging to the APP family^16^. Interestingly APP, APLP2 and ITM2B were found to form a complex interacting with GABA_b_ receptors in rodent brains^17,18^.

Despite these published data, the molecular function of ITM2B remains mostly unknown and has never been studied in the retina. To address this issue, we performed quantitative proteomics of immunopurified ITM2B complexes isolated from normal human retinal protein extracts. We used two distinct anti-ITM2B antibodies recognizing different epitopes of the protein in order to immunopurify as many ITM2B partners as possible. Gene ontology annotation with in silico tools enabled to shed light on new functional retinal pathways in which ITM2B may be involved. Interestingly, besides common protein partners purified with both antibodies, two distinct clusters of ITM2B protein interactors were identified for each of the two antibodies. To our knowledge, ITM2B interactome has only been described in rodent brains^19^, not in the retina and never in human tissues. Our findings support a specific role for ITM2B in the retina and might suggest a role in the mitochondrial homeostasis.

## Results

### Identification of ITM2B retinal interactome by quantitative proteomics

**Figure 1:**
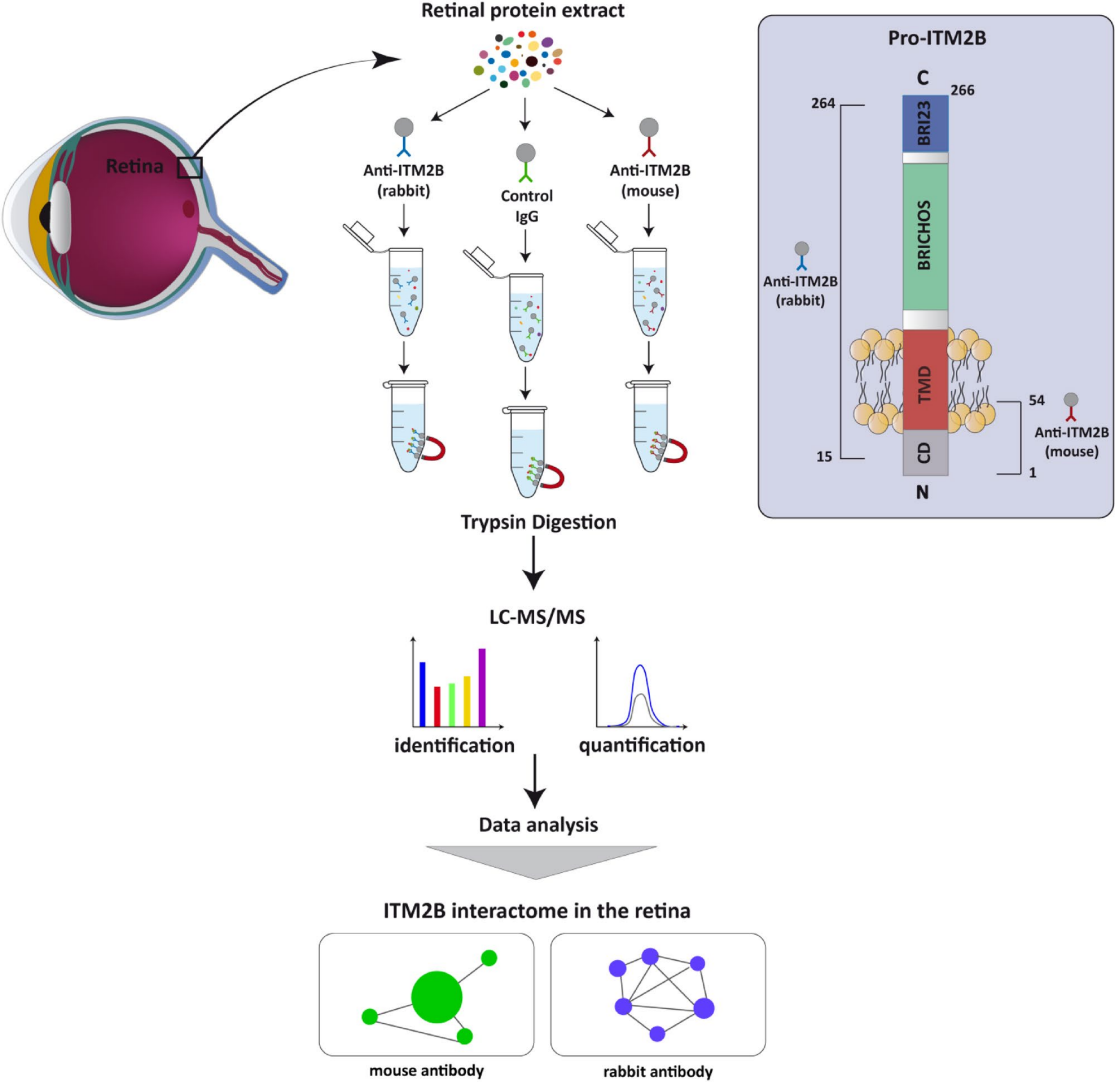
Experimental design. ITM2B is composed of several domains: CD: cytosolic domain, TMD: transmembrane domain, BRICHOS, Bri23 and two linker domains (in light grey). Two distinct anti-ITM2B antibodies were used for immunoprecipitation. The first antibody is a rabbit polyclonal antibody raised against the 15–264 aminoacidic region of ITM2B and the second antibody is a mouse monoclonal antibody raised against the N-terminal 1–54 aminoacidic region of the protein. A third unspecific IgG mouse antibody was used as negative control. After ITM2B immunoprecipitation, protein complexes were digested with trypsin and peptide mixtures were analyzed by LC–MS/MS. A label-free quantitative proteomic analysis was then performed to identify and quantify ITM2B partners.

In order to gain insight into the role of ITM2B in the retina we applied quantitative mass spectrometry-based proteomics to identify ITM2B partners. Whole proteins from an unaffected human post-mortem retina were extracted, separated in three equal samples and subjected to three independent immunoprecipitations (IP) with two anti-ITM2B antibodies raised against different epitopes of the protein and one unspecific IgG antibody. The mouse anti-ITM2B antibody binds a specific site encompassing the N-terminal 1–54 aminoacidic region of the protein, whereas the rabbit anti-ITM2B antibody binds the 15–264 aminoacidic region of ITM2B. Both antibodies immunopurified ITM2B with equal efficiency (Supplementary Fig. S1). This experimental strategy was chosen to identify as many as possible specific and functionally relevant ITM2B partners. We performed three independent experiments with one human retina (see “Materials and methods”). Immunopurified protein complexes from each retinal sample were analyzed using LC–MS/MS in triplicates. This proteomic analysis provides the first dataset of the ITM2B interactome in the retina. The workflow of the experience is depicted in Fig. 1.

Stringent filtering identified a total of 1102 proteins from immunopurified ITM2B complexes with the two anti-ITM2B antibodies. To concentrate on specific interactors, only ITM2B protein partners with a fold change > 2 (FC > 2) compared to the negative control and a p-value < 0.01 were retained. Considering only specific interactors, (FC > 2 and p-values < 0.01) a total of 457 putative ITM2B interactors were identified in the human retina (Supplementary Table S1).

The vast majority of the identified proteins are not reported as specifically expressed in the retina. Indeed, we compared our dataset with a Human Protein Atlas database list of 310 proteins highly expressed in the retina compared to other tissues. Among the 457 putative ITM2B interactors, only 9 proteins are highly expressed in the retina (Supplementary Table S2). These proteins are almost all exclusively expressed in photoreceptor cells and their associated biological processes are visual perception and photoreceptor development (Supplementary Table S3). However, we also identified interactors localized in all retinal cell types. Indeed, among 457 ITM2B potential interactors, we found tubulin beta-3 class III (TUBB3) and the neurofilament light polypeptide (NEFL) both highly expressed in retinal ganglion cells^20,21^. In addition, the well-known marker for Müller glial cells, Dickkopf-related protein 3 (DKK3)^22^, was also identified. Likewise, other proteins such as the sodium/potassium-transporting ATPase subunit beta-1 (ATP1B1)^23^ specific to amacrine cells and the septin-4 (SEPT4)^24^ specific to horizontal cells were also found (p-value < 0.05). Interestingly, we also found key proteins of the vesicular trafficking and exocytose pathways such as syntaxin binding protein 1 (STXBP1), synaptotagmin-11 (SYT11), the trafficking protein particle complex subunit 2-like protein (TRAPPC2L), Kinesin-like protein KIF21A (KIF21A) and vesicle-trafficking protein SEC22b (SEC22B). This result reinforces previous studies that reported the anterograde transport of ITM2B in the nerve terminals^15,19^.

To exhaustively identify known molecular pathways in which ITM2B may be involved, we apply gene ontology (GO) analysis to the 457 ITM2B interactors. Cellular component (GO-CC) annotations revealed protein interactome associated with organelle inner membrane, and other GO-CC terms associated more specifically with the mitochondria such as mitochondrial inner membrane, inner mitochondrial membrane protein complex, mitochondrial respirasome and mitochondrial respiratory chain complex I (Supplementary Fig. S2, Supplementary Table S4). Gene ontology biological process (GO-BP) identified interesting biological pathways such as oxidative phosphorylation and GO-BP terms associated with mitochondrial processes (cellular respiration, respiratory electron transport chain, mitochondrial respiratory chain complex I assembly, mitochondrial ATP synthesis coupled electron transport, mitochondrial electron transport/NADH to ubiquinone, and NADH dehydrogenase activity) (Supplementary Fig. S2, Supplementary Table S4). These results suggest a possible involvement of ITM2B in mitochondrial functions, in particular in cellular respiration.

### Differential interactomes identified by the anti-ITM2B mouse versus the rabbit antibody

**Figure 2:**
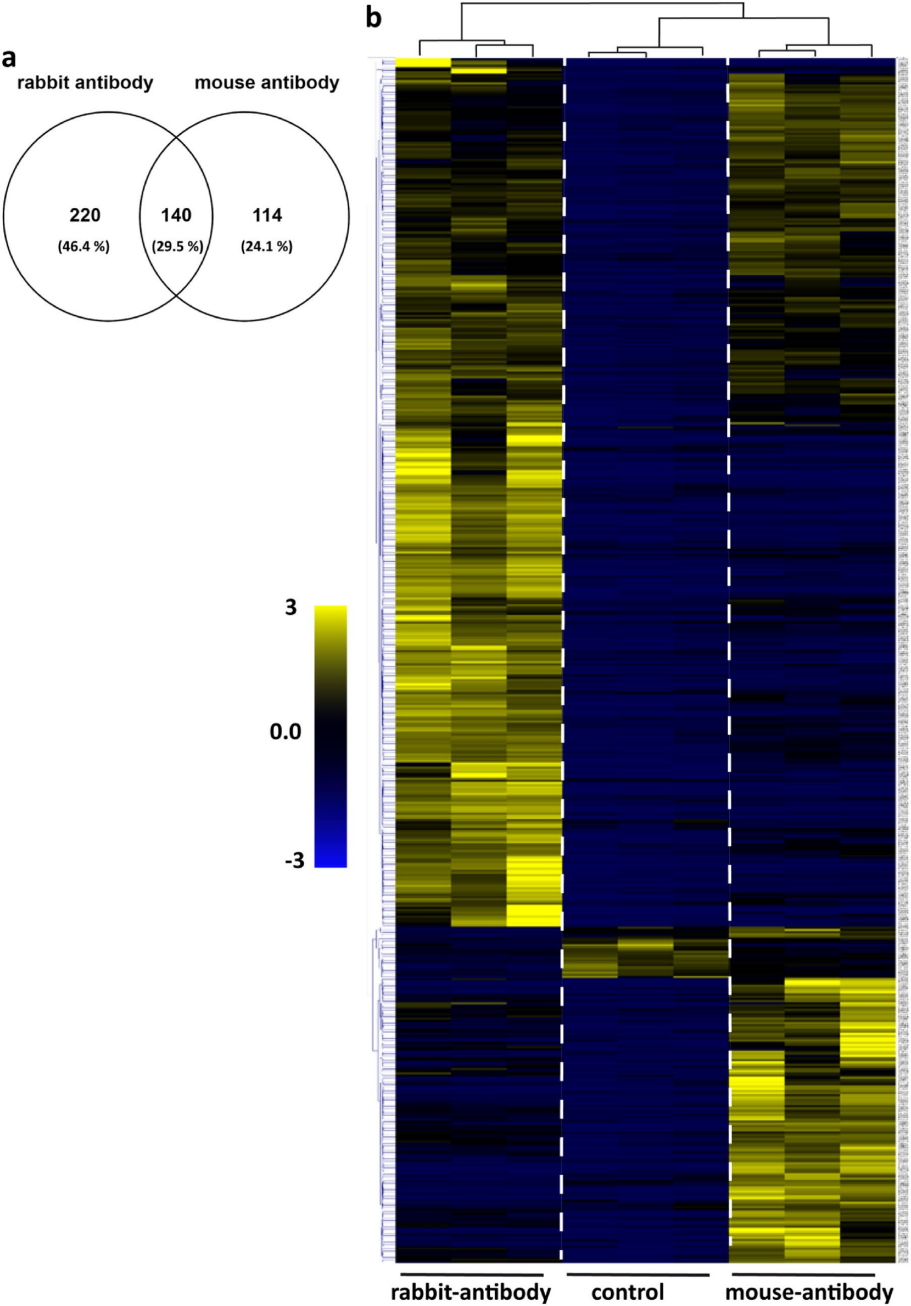
(**a**) Venn diagram showing the number of proteins purified respectively with the rabbit and the mouse antibodies. (**b**) Unsupervised hierarchical clustering and heatmap overview showing the protein abundance patterns using the two different antibodies (Multiple Experiment Viewer software, version 4.9).

We first focused on the common ITM2B partners immunopurified with both antibodies. Indeed, 254 and 360 proteins were identified respectively with the mouse and rabbit anti-ITM2B antibody (Fig. 2a). Among them, a total of 140 proteins were immunopurified with both antibodies (Fig. 2a, Supplementary Table S5). GO-CC analysis of these 140 common interactors showed proteins involved in supramolecular fiber, polymeric cytoskeletal fiber and microtubule (Supplementary Fig. S3, Supplementary Table S6). A subsequent network analysis shows an association between polymeric cytoskeletal fiber and the two other annotations (i.e. supramolecular fiber, microtubule). Only one significantly enriched GO-BP term was found: organelle localization by membrane tethering. We thus concluded that proteins with GO-BP or CC terms associated with the mitochondria were purified with only one of the two antibodies. To describe more precisely the protein complexes immunopurified by each antibody we analyzed ITM2B interactors with unsupervised hierarchical clustering. We then established a heatmap of identified proteins according to the antibodies used for protein complex purification (Fig. 2b). Beside the 140 common proteins, we also found two distinct clusters of 114 and 220 proteins specifically purified with the mouse and rabbit anti-ITM2B antibody respectively (Fig. 2b, Supplementary Fig. S4 and Fig. S5). To better characterize these two specific clusters, each dataset was studied separately.

### ITM2B interactome purified specifically with the mouse anti-ITM2B antibody

**Figure 3:**
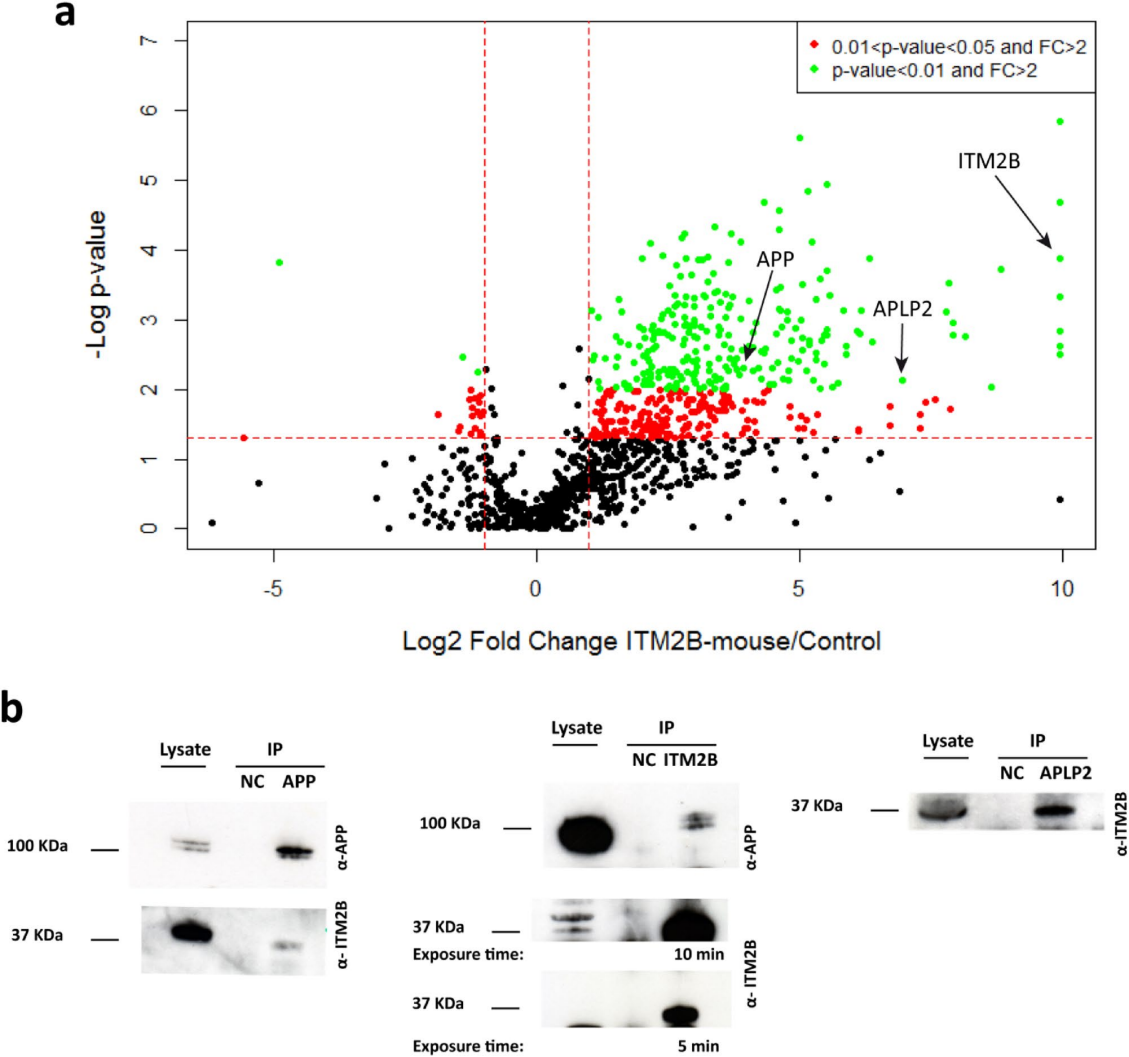
(**a**) Volcano plot with − log10 p-value vs. log2 FC (mouse anti-ITM2B/unspecific antibody). Each point represents an identified protein. Proteins with significantly different abundances (in red 0.01 < p-value < 0.05, in green p-value < 0.01) are above the horizontal red line. On the right side are the proteins enriched with the mouse anti-ITM2B antibody with a FC > 2 while on the left side are the proteins enriched with the unspecific antibody with FC > 2. (**b**) Co-immunoprecipitation of APP and APLP2 from the human retina. The IP for negative controls (NC) was performed using Dynabeads and mouse IgG (isotypic control). Raw data of Western blots are presented in Supplementary Fig. S9.

We analyzed the cluster of proteins specifically purified with the mouse antibody. A total of 440 significantly enriched proteins (FC > 2 and p-value < 0.05) were identified and are represented in the top right panel of the volcano plot (Fig. 3a). Among them, 254 statistically significant proteins were identified using a p-value < 0.01 (Fig. 3a). The specificity of the mouse anti-ITM2B antibody was rather high as illustrated by the low number of significantly enriched proteins identified by the mouse IgG unspecific antibody (top left panel). Among the significantly enriched proteins purified with the mouse anti-ITM2B antibody, we found APP and APLP2 proteins which have been previously described as ITM2B partners, notably playing a role in GABA_b_ signaling in rodent brains^17,18^. Surprisingly, no protein belonging to the GABA_b_ receptors were identified in our study, which might suggest a different role of the ITM2B–APP–APLP2 complex in the retina. We performed co-immunoprecipitation and immunoblotting experiments from human retinal protein extracts to confirm these proteomic data (Fig. 3b). To our knowledge, this is the first time that interactions between ITM2B and APP, and ITM2B and APLP2 are shown in the human retina (Fig. 3b).

**Figure 4:**
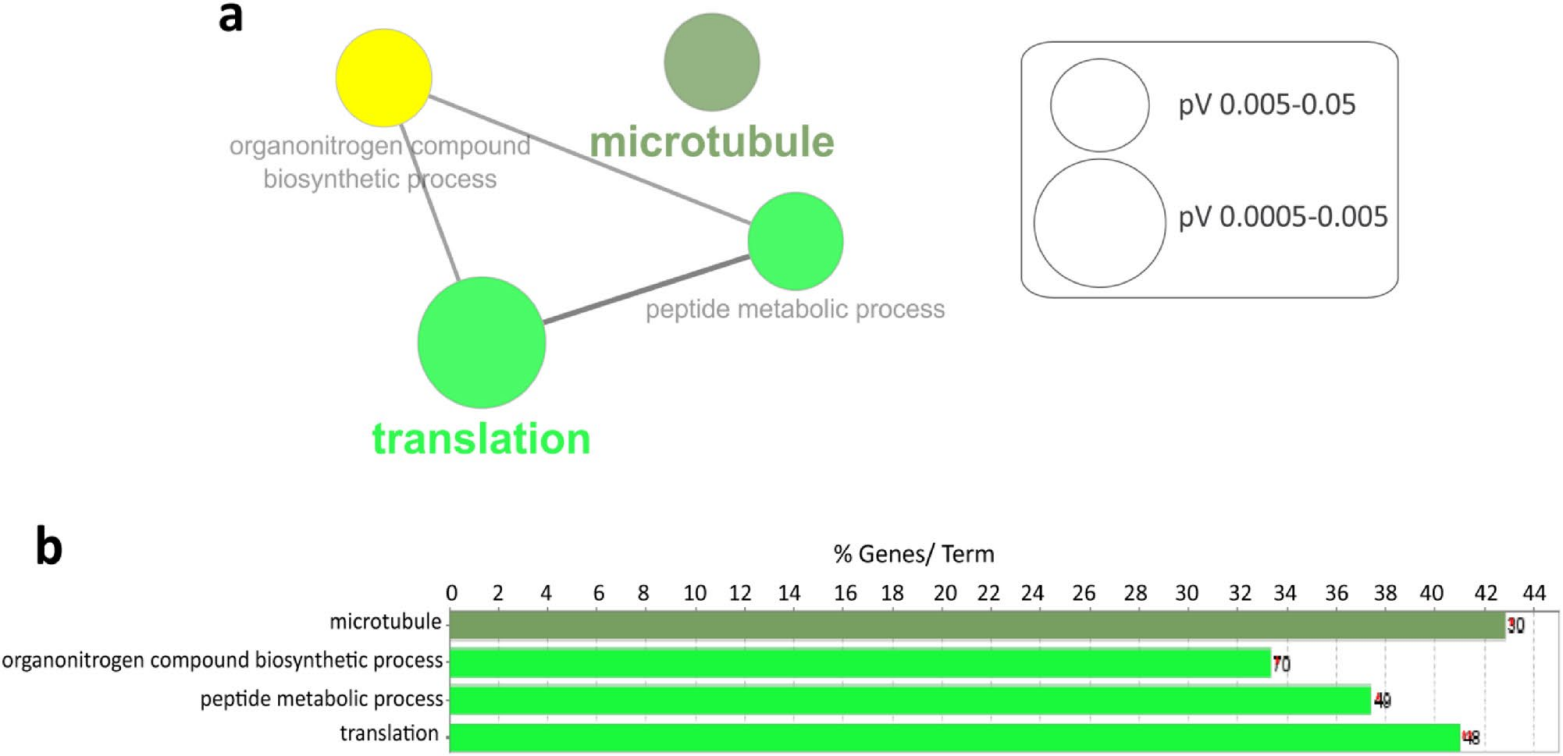
ITM2B interactome in the retina with the 254 proteins purified with the mouse antibody (FC > 2 and p-value < 0.01). (**a**) GO-term network analysis. (**b**) Functional clusters and pathways enriched within the ITM2B interactors. %Genes/Term corresponds to the proportion of genes enriched in the functional clusters. Bars with the same color belong to the same functional cluster.

As expected from the previous analyses, GO-CC annotation of the 254 specific proteins (p-value < 0.01), revealed proteins associated with microtubule (Fig. 4, Supplementary Table S7). Besides, GO analysis of ITM2B retinal interactome identified three other interesting functional pathways: organonitrogen compound, biosynthetic process, peptide metabolic process and translation (Supplementary Table S7). Subsequent network analysis shows common interactions between these last three GO-BP terms (Fig. 4a).

We then compared this proteomic dataset with the previously reported ITM2B interactome purified with the same mouse antibody from rat brains^19^. Overall, 124 proteins are in common between our dataset and the 511 rat cerebral proteins identifed in a previous study^19^ (Supplementary Table S8). The majority of these common proteins are involved in translation process including the eukaryotic translation elongation factor 1 alpha 1 (EEF1A), the ribosomal protein L12 (RPL12) and the solute carrier family 25 member 11 (SCL25A11) or involved in microtubule processes such as tubulin beta 2A (TUBB2A), tubulin beta 2B (TUBB2B) and tubulin beta-3 class III (TUBB3) (Supplementary Table S8). This comparative analysis strongly suggests that some functions of ITM2B are similar in the brain and the retina.

### ITM2B interactome purified specifically with the rabbit anti-ITM2B antibody

**Figure 5:**
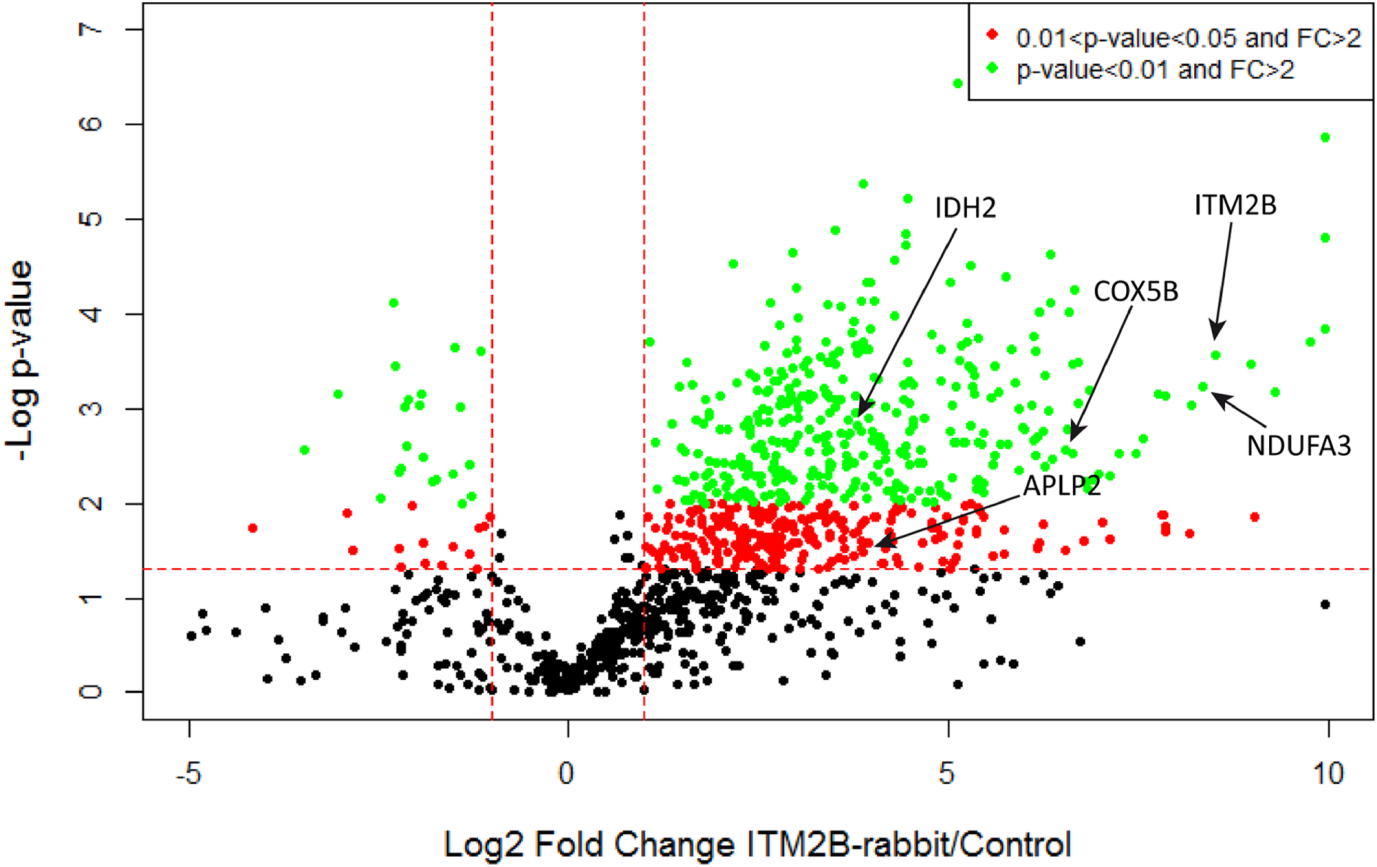
Volcano plots with − log10 p-value vs. log2 FC (rabbit anti-ITM2B/unspecific antibody). Each point represents an identified protein. Proteins with significantly different abundances (in red 0.01 < p-value < 0.05, in green p-value < 0.01) are above the horizontal red line. On the right side are the proteins enriched with the rabbit anti-ITM2B antibody with FC > 2 while on the left side are the proteins enriched with the unspecific antibody with FC > 2. IDH2, COX5B and NDUFA3 are examples of identified mitochondrial proteins.

**Figure 6:**
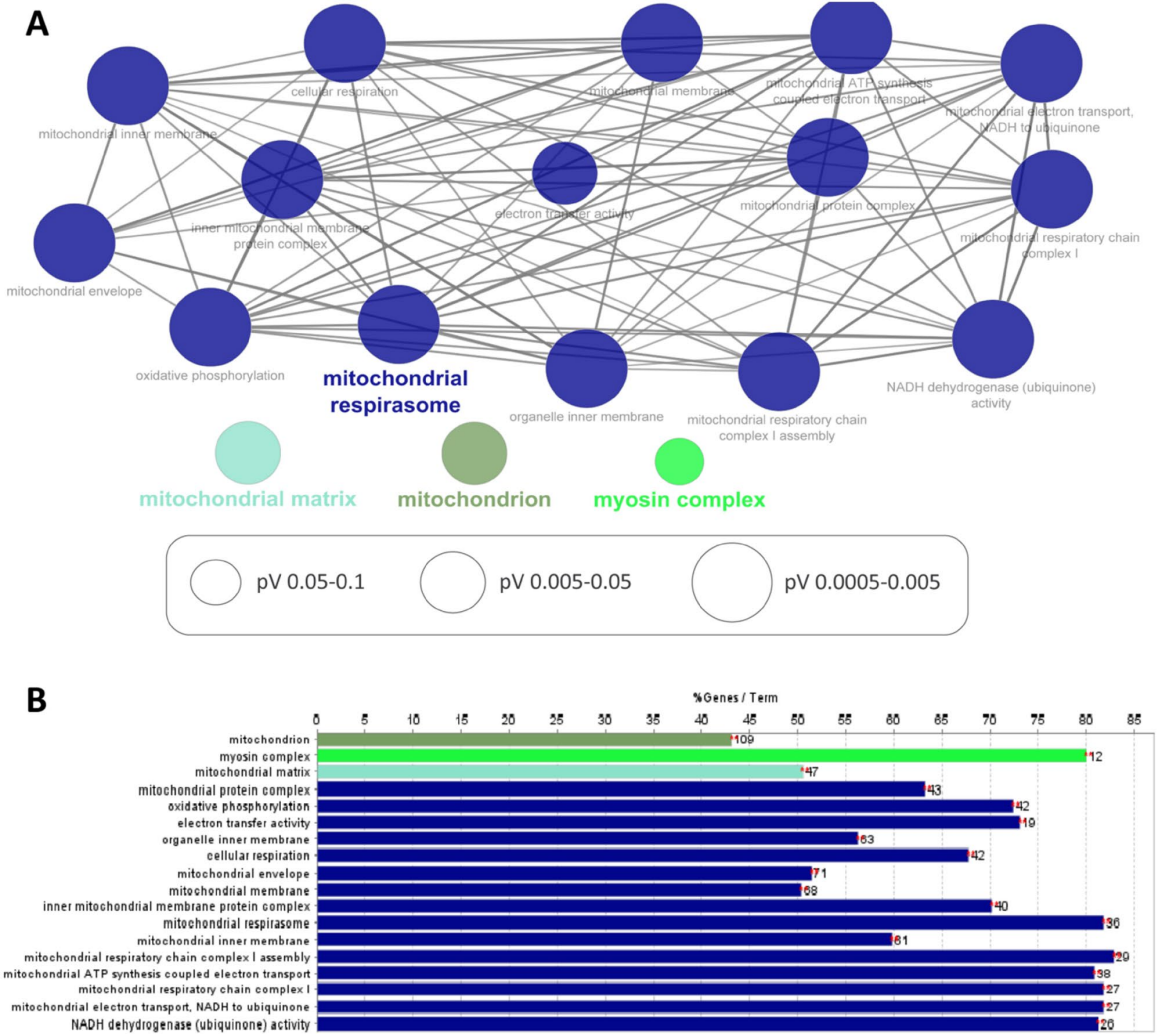
ITM2B interactome in the retina with the 360 proteins purified with the rabbit antibody (FC > 2 and p-value < 0.01). (**A**) GO-term network analysis. (**B**) Functional clusters and pathways identified with enriched proteins. %Genes/Term corresponds to the proportion of genes enriched in the functional clusters. Bars with the same color belong to the same functional cluster.

We then analyzed the cluster of proteins specifically purified by the rabbit anti-ITM2B antibody. We found a total of 615 enriched proteins (FC > 2 and p-value < 0.05). Among these proteins, 360 were identified with a p-value < 0.01 and are shown in the top right panel of the volcano plot (Fig. 5). We performed GO analysis on these proteins and the main biological pathways that we found are involved in mitochondrial processes, such as oxidative phosphorylation, electron transfer activity, cellular respiration, mitochondrial respiratory chain complex I assembly, mitochondrial ATP synthesis coupled electron transport, mitochondrial electron transport, NADH to ubiquinone and NADH dehydrogenase activity (Fig. 6, Supplementary Table S9). Furthermore, GO-CC annotations refer to mitochondria including mitochondrion, mitochondrial matrix, mitochondrial protein complex, and mitochondrial envelope (Fig. 6, Supplementary Table S9). These annotations have never been reported so far and suggest a new unsuspected function of ITM2B associated with the mitochondria.

To strengthen this new result, we checked the interaction between ITM2B and COX5B one of the mitochondrial proteins identified in this study. We found that immunoprecipitated endogenous COX5B interacts with overexpressed ITM2B in HEK 293 cells (Supplementary Fig. S6). Moreover, by immunohistochemistry on human retina sections, we observed co-localization of ITM2B not only with COX5B but also with FKBP8 and to a minor extent with ATP5F1B, two other mitochondrial proteins identified in this study (Supplementary Fig. S7). The poor co-localization between ITM2B and ATP5F1B is most probably the consequence of the suboptimal detection of the endogenous ITM2B observed with the rabbit anti-ITM2B antibody (Supplementary Fig. S7, ITM2B staining, panels a and b). Altogether these data indicate an involvement of ITM2B in mitochondrial functions.

Additionally, GO-CC terms revealed significantly enriched proteins in the myosin complex related to the cytoskeleton, distinct from the microtubule GO-BP annotation found with the mouse anti-ITM2B antibody (Fig. 6, Supplementary Table S9).

Unlike the mouse anti-ITM2B antibody, the rabbit anti-ITM2B has never been used before for affinity-purification-MS experiments. However, the cluster of specific proteins purified with the rabbit anti-ITM2B antibody was also compared with the rat cerebral ITM2B interactome. Among the 150 proteins found in common, we observed proteins associated with the cytoskeleton organization including the adducing 1 (ADD1), the plectin (PLEC) and the vimentin (VIM) (Supplementary Table S10). This comparative analysis supports the reliability of the ITM2B interactome identified with the rabbit antibody.

### Different ITM2B transcripts are expressed in the human retina

**Figure 7:**
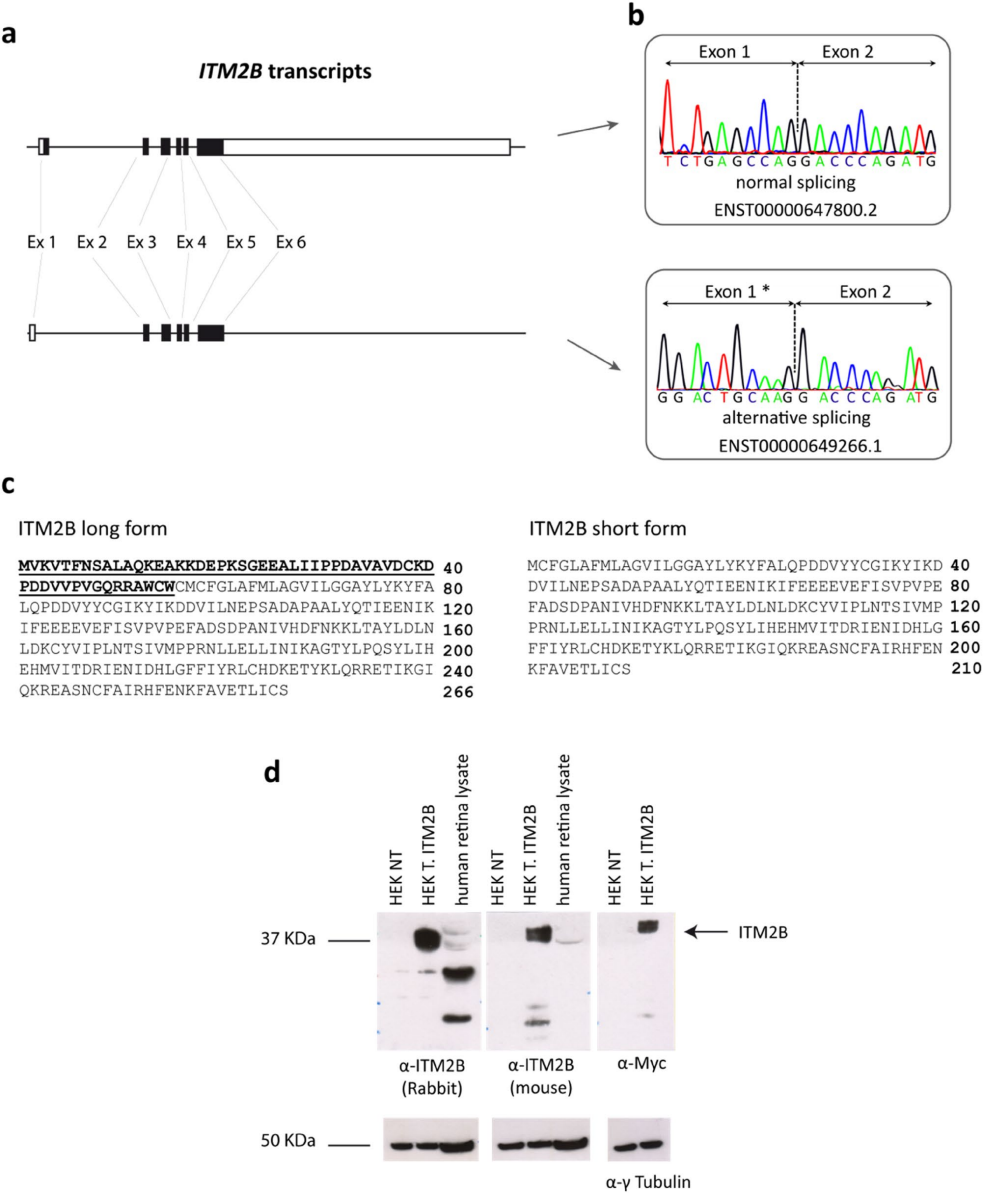
Identification of ITM2B alternative transcripts in the human retina. (**a**) Different ITM2B splice variants. (**b**) Identification of ITM2B alternative splicing in the human retina. (**c**) Amino acid sequence for ITM2B long isoform and predicted amino acid sequence for ITM2B short isoform. (**d**) Western blots on HEK 293 cells and retinal protein extract, *NT* non transfected, *T* transfected with ITM2B, anti-γ-tubulin is used as a loading control. Raw data of Western blots are presented in Supplementary Fig. S10.

As described before, common interactors as well as distinct clusters of proteins were identified using the two antibodies. One possible explanation to these distinct clusters is given by the difference in the epitopes against which each antibody was raised (Fig. 1). Interestingly, a new human ITM2B alternative transcript was recently annotated in Ensembl (ENST00000649266.1). This transcript harbors a translation initiation codon in exon 2 and is predicted to generate a shorter ITM2B protein (210 amino acids). For the rest of this manuscript, this isoform will be named short form whereas the canonical 266-amino acid protein will be the long form (transcript ENST00000647800.2). Based on the distinct epitopes used to generate the antibodies, the rabbit anti-ITM2B antibody would recognize both the short and the long forms whereas the mouse anti-ITM2B antibody would recognize only the long one. To check the expression of both transcripts in the human retina, we performed PCR experiments on human retinal cDNA using specific primers for each isoform followed by Sanger sequencing. Both *ITM2B* transcripts were identified in the human retina (Fig. 7). We thus speculate that the different ITM2B interactomes isolated by each of the two antibodies might be the consequence of the unique ability of the rabbit anti-ITM2B antibody to immunopurify the long as well as the short isoform of ITM2B. Indeed, as shown in Fig. 7, the rabbit anti-ITM2B antibody, while detecting the canonical 266-amino acid protein as the mouse antibody, also recognizes at least three other proteins in total human retinal protein lysate.

## Discussion

ITM2B is an intriguing protein involved in different cerebral and retinal disorders. Three distinct autosomal dominant mutations in this gene are associated with two different early onset severe forms of dementia (FBD and FDD)^5,8^ and with one peculiar retinal dystrophy^3,10^. Interestingly, there is no report of retinal degeneration in patients affected with ITM2B-related dementia but early onset cataract and a microangiopathy (FDD)^23^. Reciprocally patients carrying the mutation leading to the retinal dystrophy do not develop dementia. These clinical data support the idea that ITM2B could have different roles in the brain and in the retina. All previous studies focused on ITM2B functions in the brain. More specifically, ITM2B is known to interact with APP and plays a role in Aβ cerebral metabolism. In addition, ITM2B has been involved in GABA_b_, glutamate receptor signaling pathways and neurite outgrowth^15,17,18,24^. In this study we aimed at identifying the ITM2B interactome in the retina to unravel new biological pathways of this protein.

A total of 457 proteins were identified by quantitative proteomics of human retinal immunopurified ITM2B complexes (Supplementary Table S1). Among them, different retinal cell markers were identified suggesting that ITM2B acts ubiquitously in retinal tissue. Furthermore, our group previously showed that *ITM2B* is expressed in different retinal cells of the inner nuclear layer and in retinal ganglion cells^3,10^. The proteomic analysis identified 8 proteins highly expressed in photoreceptor cells. Since photoreceptors are a major cell type in the retina, there may be a possibility that highly abundant photoreceptor proteins were nonspecifically captured. However, this finding may also indicate that ITM2B is present in photoreceptor cells. Furthermore, ITM2B immunostaining using the mouse antibody showed ITM2B localization in the photoreceptor layer (Supplementary Fig. S7). This is supported by transcriptomic data which suggests the ubiquitous expression of ITM2B in human retina^25,26^.

Interestingly, APP and APLP2 previously reported to interact with ITM2B in the brain^17,18^ were also found in our retinal dataset^17,18^. APLP2 is particularly interesting since its deletion in the mouse retina induces a phenotype similar to the retinal dystrophy observed in subjects carrying the ITM2B c.782A>C, p.Glu261Ala mutation notably with similar electroretinogram alterations^3,27^. The ITM2B-retinal dystrophy mutation could lead to a loss of interaction between APLP2 and the mutant ITM2B inducing a similar phenotype as the *Aplp2*^−/−^ mice. Further experiments to support this hypothesis. In addition, more than 40% of the ITM2B interactors were also identified in the previous rat cerebral ITM2B interactome^19^. All these data indicate that ITM2B has overlapping but also specific functions in the brain and in the retina.

Gene Ontology analyses of the whole retinal interactome revealed several terms associated to cytoskeleton, translation and more surprisingly mitochondria. While the functional involvement of ITM2B in cytoskeleton and vesicular trafficking processes has already been demonstrated^17–19^, the link between ITM2B and mitochondrial functions has never been reported so far.

The human retinal ITM2B interactome was identified by combining the protein complexes isolated using two different anti-ITM2B antibodies. GO-BP analysis of the ITM2B interactome specifically purified with the mouse antibody revealed that beyond microtubule association, most functional pathways are linked to protein translation, organonitrogen compound biosynthetic process and peptide metabolism. These two last functional annotations were found neither in the whole dataset analysis nor in the previous published rat cerebral interactome. ITM2B has never been associated with metabolic processes and this result might suggest a new and specific role of ITM2B in the retina. We next analyzed the ITM2B specific interactome isolated with the rabbit antibody. Most of the GO-BP terms indicate that ITM2B might be involved in mitochondrial functions. While this annotation did not emerge in the previous published proteomic analysis, several mitochondrial proteins were also identified in the ITM2B interactome purified with the mouse antibody in the human retina and in the rat brain. This result strengthens the reliability of these new data. The putative role of ITM2B in the retinal mitochondrial homeostasis is of particular interest while studying pathogenic mechanisms associated with ITM2B mutations. Indeed, among other retinal abnormalities, affected subjects carrying the retinal dystrophy-related ITM2B missense mutation exhibit an early onset ganglion cell loss^3,10^. Mitochondrial dysfunction is a common mechanism underlying optic nerve diseases including primary ganglion cell disorders^28^. This newly identified role of ITM2B in the mitochondria may shed light on the early ganglion cell loss observed in subjects carrying the missense mutation. To date, we do not know if the link between ITM2B and the mitochondria is specific to the retina since the rabbit anti-ITM2B antibody was never used in previous proteomic studies. Therefore, we cannot rule out the possible involvement of ITM2B in cerebral mitochondrial homeostasis. Indeed, several mitochondrial proteins identified in the present study, such as the cytochrome c oxidase subunit 5B (COX5B), the cytochrome c oxidase subunit 7A-related protein (COX7A2L), the mitochondrial 2-oxoglutarate/malate carrier protein (SLC25A11) and the phosphate carrier protein (SLC25A3) have been also identified in the rat cerebral cortex ITM2B interactome^19^.

The diversity of GO-BP terms associated to the ITM2B interactomes may reflect a difference in the biochemical nature of the protein complexes immunopurified with each antibody. Indeed, ITM2B undergoes proteolytic cleavages and each antibody preferentially binds to different fragments of the protein and purify distinct complexes. For instance, ITM2B is cleaved at amino acid 243 by a furin-like protease releasing Bri23, a 23-amino acid peptide. Bri23 is potentially immunopurified only by the rabbit antibody and may represent the mitochondrial-specific ITM2B bait. Alternatively, the two antibodies could purify different protein isoforms with different functions. Additionally, this could also be extended to the differences in ITM2B localization observed when performing immunostainings on human retinal sections using the two antibodies. Of note, a new human ITM2B alternative transcript was recently annotated. Its predicted coding sequence would generate a 210-amino acid protein which would not be recognized by the mouse antibody and could represent, as for Bri23, the mitochondrial-specific ITM2B bait. Interestingly, in a murine T cell line, a shorter form of ITM2B (ITM2Bs) involved in the mitochondria homeostasis was previously reported^29^. This shorter form corresponds to the one recently annotated in human and would have a pro-apoptotic effect by interacting with Bcl-2. Interestingly, Bcl-2-associated transcription factor 1 (BCLAF1) was also found in the human retinal ITM2B interactome. To date, we were able to show that the transcript of this shorter isoform is present in the human retina. Unfortunately, it will be very difficult to study this isoform at the protein level due to the lack of specific antibody. Taken together, these data show that the use of antibodies directed against distinct epitopes of a protein may allow the identification of different protein interactors helping to unveil still unknown cellular functions.

Recently, our group generated induced pluripotent stem cell lines (iPSCs) from a subject affected with the ITM2B-related retinal dystrophy and his unaffected sibling^30^. Further investigations using retinal organoids derived from these iPSCs will help to better characterize the ITM2B interactome during retinal development and the impact of the ITM2B-retinal dystrophy mutation on protein interactors. Moreover, it will give new insights into the role of ITM2B in mitochondrial homeostasis.

## Materials and methods

### Preparation of human retina samples

Postmortem human ocular globes from donors were acquired from the School of Surgery (Ecole de Chirurgie, Assistance Publique Hôpitaux de Paris, Paris, France) and from the Laboratory of Anatomy, (Faculty of Medicine of St-Etienne, France). Experiments on postmortem human retina were performed according to the National regulations, as well as the guidelines of the Declaration of Helsinki. The protocol was approved by the IRBs of the School of Surgery and Faculty of Medicine of St-Etienne. The authorization for the use of human samples for research at the Institut de la Vision is registered as CODECOH DC-2015-2400.

One post-mortem human retina was obtained from an 85-year old man with no apparent history of ophthalmic disorder. The entire retina without the macula was put in liquid nitrogen and conserved in − 80 °C. The retina was then thawed on ice and gently cut in smaller pieces with surgical scissors to homogenize the sample before protein extraction. Total proteins were extracted from the human retina using 1.8 ml lysis buffer (50 mM Tris pH 7.5, 150 mM NaCl, 1% Triton 100 X) containing protease and phosphatase inhibitors (1% Phosphatase inhibitor cocktail 2 and 3 Sigma, 1% Protease inhibitor cocktail Merck, Darmstadt, Germany). The mix of lysis buffer and human retina was kept on ice during 30 min with vortexing and pipetting every 10 min. After centrifugation 13,000 rpm (15,800 RCFs) 10 min, the supernatant containing the proteins was transferred to a new tube. All the extracted proteins (0.97 µg/µl) were used for immunoprecipitation.

Extracted proteins from other entire normal post-mortem human retinas respectively obtained from a 97-year old man, an 84-year old woman and a 90-year old man [0.90–1.5 µg/µl] were used in order to validate ITM2B interactions with its partners (see “Interaction validation by immunoblotting”).

### Immunoprecipitation

We used Dynabead protein G (Thermo Fisher Scientific, Waltham, USA) to perform immunoprecipitation. We chose two different anti-ITM2B antibodies: one raised in rabbit (14.88 µg; PA531441 Thermo Fisher Scientific), the other raised in mouse (6 µg; SC-374362 Santa Cruz Biotechnology, Dallas, USA) and one mouse IgG (6 µg; G3A1, Cell Signaling Technology, Leiden, Netherlands) as a control. The procedure below was performed three independent times to obtain triplicates. A volume of 50 µl of beads solution was incubated with the 3 different antibodies and 500 µl PBS 1 × 0.02% Tween 20 for each antibody for 1.30 h at 4 °C under shaking. After one wash with 500 µl PBS 0.02% Tween 20, 190 µl of human retina protein extract (0.97 µg/µl) were applied to the antibody-Dynabeads mix and incubated overnight at 4 °C under shaking (for each condition). The immunoprecipited proteins were then washed twice with 500 µl of PBS 0.02% Tween 20 and one last time with 500 µl of PBS 1 ×.

### LC–MS/MS analyses

Proteins on beads from 3 experimental conditions (control, mouse antibody, rabbit antibody) were digested in biological triplicates overnight at 37 °C by sequencing grade trypsin (12.5 µg/ml; Promega Madison, WI, USA) in 20 µl of 25 mM NH_4_HCO_3_. Peptides were desalted using ZipTip µ-C18 Pipette Tips (Thermo Fisher Scientific). Peptide mixtures were analyzed by a Q-Exactive Plus coupled to a Nano-LC Proxeon 1000 (both from Thermo Fisher Scientific). Peptides were separated by chromatography as performed previously^31^. In brief, the following parameters were used: Acclaim PepMap100 C18 pre-column (2 cm, 75 μm i.d., 3 μm, 100 Å), Pepmap-RSLC Proxeon C18 column (50 cm, 75 μm i.d., 2 μm, 100 Å), 300 nl/min flow rate, a 98 min gradient from 95% solvent A (water, 0.1% formic acid) to 35% solvent B (100% acetonitrile, 0.1% formic acid). Peptides were analyzed in the Orbitrap cell, at a resolution of 70,000, with a mass range of *m/z* 375–1500. Fragments were obtained by higher-energy collisional dissociation (HCD) activation with a collisional energy of 28%. MS/MS data were acquired in the Orbitrap cell in a Top20 mode, at a resolution of 17,500.

### Quantification of protein abundance variations

Label-free relative quantification was performed in a Between-Subject Study Design using Progenesis-Qi software 4.1 (Nonlinear Dynamics Ltd, Newcastle, UK). For the identification step, all MS and MS/MS data were processed with the Proteome Discoverer software (Thermo Fisher Scientific, version 2.2) coupled to the Mascot search engine (Matrix Science, version 2.5.1). The mass tolerance was set to 7 ppm for precursor ions and 0.05 Da for fragments. The maximum number of missed cleavages was limited to two for the trypsin protease. As performed before^31^, the following variable modifications were allowed: oxidation (Met), phosphorylation (Ser, Thr, and Tyr), acetylation (Protein N-term). The SwissProt database (02/2017) with the *Homo sapiens* taxonomy was used for the MS/MS identification step. Peptide identifications were validated using a 1% FDR (False Discovery Rate) threshold calculated with the Percolator algorithm. Protein identifications were validated if at least two unique peptides per protein were identified. Protein abundance measurements were calculated according to the Hi-3 label-free quantification method. Protein variations were validated if their calculated p-values were under 0.01.

### In silico analyses and statistics

The list of the 310 proteins highly expressed in the human retina was obtained from the Human Protein Atlas (available online: http://www.proteinatlas.org). Venn Diaphragms were generated using Venny online tool^32^ (version 2.1). Hierarchical clustering and heatmap were performed by the MEV (Multiple Experiment Viewer software) software (version 4.9) with a Pearson correlation^33^ and the average linkage clustering method. Ontology term enrichment and visualization were obtained using the ClueGo plug-in (version 2.5.6)^34^ within the Cytoscape software (version 3.7.1). The following parameters were used for the GO term enrichment analyses: (i) right sided hypergeometric test with a Benjamini–Hochberg correction with a minimum p-value threshold of 0.01, (ii) reference set with all identified proteins in concerned proteomic experiments with at least 2 unique identified peptides, (iii) GO tree interval from 4 to 8, (iv) GO term fusion activated, (v) GO terms process, function and component from EBI, UniProt and GOA. Volcano plots representing protein abundance variations between control condition and specific antibodies according to p-values were generated with R (version 3.6.1).

### Interaction validation by immunoblotting

To validate the interaction between ITM2B and its partners we performed co-immunoprecipitation and Western blot experiments using protein extracts from normal human retinas. Proteins from human retinal lysate and eluted from the co-immunoprecipitation assays were resolved on 4–12% gradient SDS-PAGE and transferred onto nitrocellulose membranes. Additional co-immunoprecipitation assays were performed using other antibodies (see Table 1). After transfer, membranes were incubated with blocking buffer PBST (PBS 1 ×, 0.05% Tween 20) with 5% non-fat powder milk and incubated with the primary antibodies (Table 1) overnight at 4 °C with shaking. The membranes were then washed three times for 10 min with PBST and incubated for 1 h at room temperature with horseradish peroxidase (HRP)-linked secondary antibodies (Table 1). Finally, the membranes were washed three times for 10 min with PBST and enhanced chemiluminescence-based system (ECL Plus, Thermo Fisher Scientific) was used for protein detection.

**Table 1:** List of primary and secondary antibodies used for immunoprecipitation and Western blot.

Antigen	Species	Dilution	Source
ITM2B	Mouse	IP: 6 µg; WB: 1/500	Santa Cruz (sc-374362)
	Rabbit	WB: 1/1000	Thermo Fisher Scientific (PA531441)
APP	Rabbit	IP: 5 µg; WB: 1/1000	Merck (HPA001462)
	Mouse	WB: 1/500	Merck (MAB348)
APLP2	Mouse	IP: 10 µg	Merck (SAB1410669)
		WB: 1/500	
COX5B	Rabbit	IP: 2 µg; WB: 1/500	Thermo Fisher Scientific (PA5-96189)
Myc	Mouse	WB: 1/500	Roche (11667149001)
γ Tubulin	Mouse	WB: 1/10,000	Merck (T6557)
IgG isotype control	Mouse	According to the specific antibody used	Cell Signaling (5415)
IgG isotype control	Rabbit	According to the specific antibody used	Cell Signaling (3900)
Mouse HRP	Donkey	WB: 1/20,000	Jackson ImmunoResearch (715-035-150)
Rabbit HRP	Goat	WB: 1/20,000	Jackson ImmunoResearch (111-035-003)

*IP* immuno-precipitation, *WB* western blot, *HRP* horseradish peroxidase.

### HEK transfection

The GripTite 293 MSR Cells (Thermo Fisher Scientific) were transfected with a ITM2B expression vector (pBudCE4_1ITM2B plasmid^3^) using lipofectamine 2000 as transfection reagent (Invitrogen, Carlsbad, Czech Republic) following the standard manufacturer’s protocol. The expression vector was designed to generate a fusion protein of ITM2B with a Myc-tag sequence placed after the ATG initial codon of the cDNA. The ITM2B sequence NM_021999.4.

### PCR and Sanger sequencing

Specific forward and reverse primers were designed for both forms (1shortF: 5′gagcttcctccacattggtc3′ and 1shortR: 5′caattgtctggtagagagcag3′; longF: 5′tgacgttcaactccgctctg3′ and longR: 5′catcatctgggtccttgcagt3′), (ENST00000649266.1). PCR was performed using Human Retina QUICK-Clone cDNA (TAKARA, Kusastu, Japan) and Q5 High-Fidelity 2 × Master Mix (New England Biolabs, Ipswich, USA) (Supplementary Table S11 and Supplementary Table S12). Specificity of all PCR products was first verified by electrophoresis on 1% agarose gel and subsequently by Sanger sequencing. Purification of PCR products was done on agarose gel for the short form using PCR clean-up Gel extraction (Macherey-Nagel, Düren, Germany) following manufacturer’s recommendations.

## Supplementary Information

Supplementary Information.

## Data Availability

The mass spectrometry proteomic data have been deposited to the ProteomeXchange Consortium via the PRIDE^35^ partner repository with the dataset identifier PXD020088. All other data generated or analyzed during this study are included in this published article (and its Supplementary Information files).
