# Supplementary material for: First identification of ITM2B interactome in the human retina
Source: Sci Rep. 2021 Aug 26;11:17210. doi: 10.1038/s41598-021-96571-6 (PMC8390696; doi:10.1038/s41598-021-96571-6)
Supplement: Supplementary file 1 — Supplementary Information. [file 41598_2021_96571_MOESM1_ESM.docx]

***Supplementary data**

**First identification of ITM2B interactome in the human retina**

J. Wohlschlegel^1^, M. Argentini^1^, C. Michiels^1^, C. Letellier^1^, V. Forster^1^, C. Condroyer^1^, Z. He^2^, G. Thuret^2,3^, C. Zeitz^1^, T. Léger^4, 5^, I. Audo^1, 6,7*^

1. Sorbonne Université, INSERM, CNRS, Institut de la Vision, 17 rue Moreau, F-75012 Paris, France

2. Corneal Graft Biology, Engineering and Imaging Laboratory, Health Innovation Campus, Faculty of Medicine, Jean Monnet University, Saint-Etienne, France.

3. Department of Ophthalmology, University Hospital, Saint-Etienne, France.

4. Mass Spectrometry Laboratory, Institut Jacques Monod, UMR 7592, Université Paris Diderot, CNRS, Sorbonne Paris Cité, F-75205 Paris, France.

5. Univ Rennes, Inserm, EHESP, Irset (Institut de recherche en santé, environnement et travail) – UMR_S 1085, F-35000 Rennes, France
6. CHNO des Quinze-Vingts, INSERM-DGOS CIC 1423, 28 rue de Charenton, F-75012 Paris, France

**7. Department of Genetics, UCL-Institute of Ophthalmology, 11–43 Bath Street, London EC1V 9EL, UK**

**Corresponding author:** Isabelle Audo, MD, PhD, Institut de la Vision, 17 Rue Moreau, Paris, France, 75012. Mail: isabelle.audo@inserm.fr

**
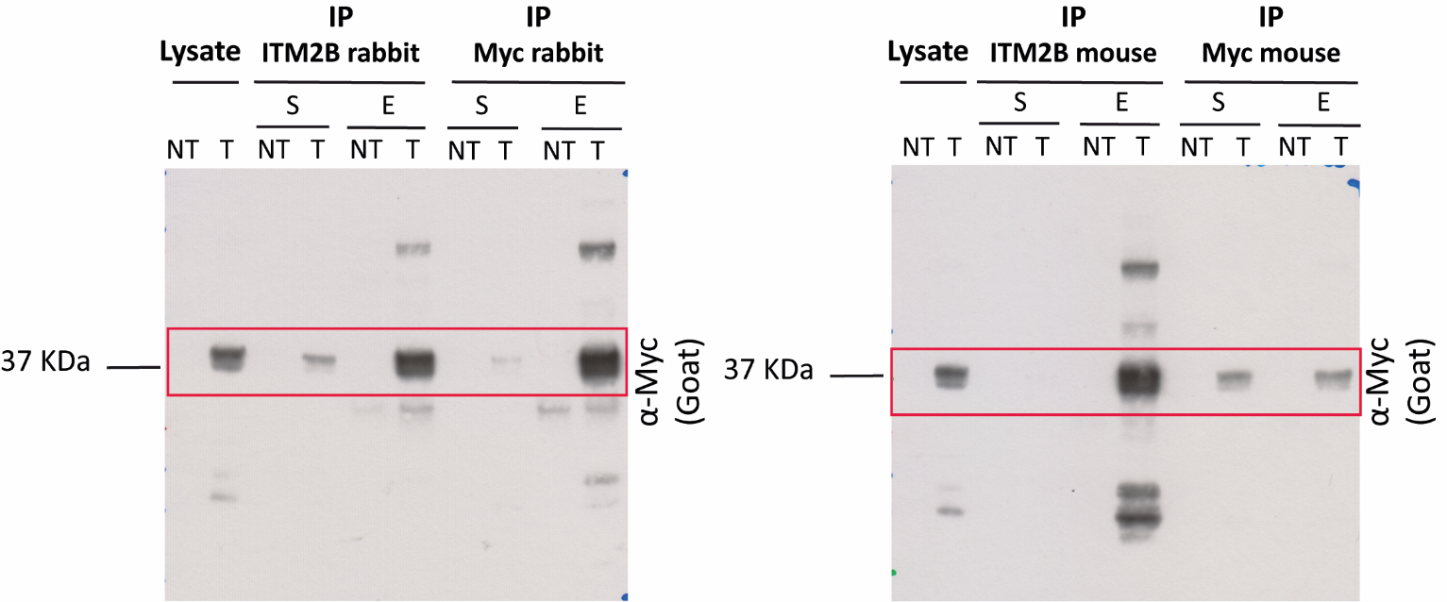
**

**Supplementary Figure S1.** Validation of anti-ITM2B antibodies using protein extracts from HEK 293 cells transiently transfected with an ITM2B-Myc-tag expression plasmid. Red boxes highlight the regions of ITM2B (37 kDa). Lysate: HEK-293 protein extract; IP: immunoprecipitation; E: Eluate; S: Supernatant; NT: non transfected; T: transfected with ITM2B; α-: indicates the antibody used for the staining.

**Supplementary Table S1**. Curated list representing 457 proteins identified by LC-MS/MS and purified with the mouse and/or the rabbit anti-ITM2B antibodies (FC >2 and p-value<0.01).

| **Uniprot accession** | **Gene name** | **Protein name** |
| --- | --- | --- |

| P01023 | *A2M* | alpha-2-macroglobulin |
| --- | --- | --- |
| P61221 | *ABCE1* | ATP binding cassette subfamily E member 1 |
| Q8NFV4 | *ABHD11* | abhydrolase domain containing 11 |
| Q9NYB9 | *ABI2* | abl interactor 2 |
| Q9H845 | *ACAD9* | acyl-CoA dehydrogenase family member 9 |
| P11310 | *ACADM* | acyl-CoA dehydrogenase medium chain |
| P45954 | *ACADSB* | acyl-CoA dehydrogenase short/branched chain |
| Q9UKV3 | *ACIN1* | apoptotic chromatin condensation inducer 1 |
| P24666 | *ACP1* | acid phosphatase 1 |
| Q4G176 | *ACSF3* | acyl-CoA synthetase family member 3 |
| O95573 | *ACSL3* | acyl-CoA synthetase long chain family member 3 |
| P60709 | *ACTB* | actin beta |
| P68032 | *ACTC1* | actin alpha cardiac muscle 1 |
| P61160 | *ACTR2* | actin related protein 2 |
| Q08828 | *ADCY1* | adenylate cyclase 1 |
| P35611 | *ADD1* | adducin 1 |
| Q9UEY8 | *ADD3* | adducin 3 |
| Q9UPQ3 | *AGAP1* | ArfGAP with GTPase domain, ankyrin repeat and PH domain 1 |
| P23526 | *AHCY* | adenosylhomocysteinase |
| P27144 | *AK4* | adenylate kinase 4 |
| P02768 | *ALB* | Albumin |
| P49418 | *AMPH* | Amphiphysin |
| Q12955 | *ANK3* | ankyrin 3 |
| O95782 | *AP2A1* | adaptor related protein complex 2 subunit alpha 1 |
| Q96CW1 | *AP2M1* | adaptor related protein complex 2 subunit mu 1 |
| Q06481 | *APLP2* | amyloid beta precursor like protein 2 |
| P05067 | *APP* | amyloid beta precursor protein |
| P55087 | *AQP4* | aquaporin 4 |
| Q8N6H7 | *ARFGAP2* | ADP ribosylation factor GTPase activating protein 2 |
| Q15052 | *ARHGEF6* | Rac/Cdc42 guanine nucleotide exchange factor 6 |
| P40616 | *ARL1* | ADP ribosylation factor like GTPase 1 |
| P36404 | *ARL2* | ADP ribosylation factor like GTPase 2 |
| Q9NVJ2 | *ARL8B* | ADP ribosylation factor like GTPase 8B |
| P59998 | *ARPC4* | actin related protein 2/3 complex subunit 4 |
| Q8NBU5 | *ATAD1* | ATPase family AAA domain containing 1 |
| Q9NVI7 | *ATAD3A* | ATPase family AAA domain containing 3A |
| Q676U5 | *ATG16L1* | autophagy related 16 like 1 |
| P13637 | *ATP1A3* | ATPase Na+/K+ transporting subunit alpha 3 |
| Q01814 | *ATP2B2* | ATPase plasma membrane Ca2+ transporting 2 |
| P23634 | *ATP2B4* | ATPase plasma membrane Ca2+ transporting 4 |
| P25705 | *ATP5F1A* | ATP synthase F1 subunit alpha |
| P06576 | *ATP5F1B* | ATP synthase F1 subunit beta |
| P36542 | *ATP5F1C* | ATP synthase F1 subunit gamma |
| P18859 | *ATP5PF* | ATP synthase peripheral stalk subunit F6 |
| Q93050 | *ATP6V0A1* | ATPase H+ transporting V0 subunit a1 |
| P61421 | *ATP6V0D1* | ATPase H+ transporting V0 subunit d1 |
| P38606 | *ATP6V1A* | ATPase H+ transporting V1 subunit A |
| P21281 | *ATP6V1B2* | ATPase H+ transporting V1 subunit B2 |
| Q9Y5K8 | *ATP6V1D* | ATPase H+ transporting V1 subunit D |
| O43505 | *B4GAT1* | beta-1,4-glucuronyltransferase 1 |
| Q9UL15 | *BAG5* | BAG cochaperone 5 |
| P46379 | *BAG6* | BAG cochaperone 6 |
| Q9NYF8 | *BCLAF1* | BCL2 associated transcription factor 1 |
| P38398 | *BRCA1* | BRCA1 DNA repair associated |
| P35613 | *BSG* | basigin (Ok blood group) |
| P30042 | *C21orf33* | ES1 protein homolog, mitochondrial |
| Q9Y426 | *C2CD2* | C2 calcium dependent domain containing 2 |
| P01024 | *C3* | complement C3 |
| P0C0L4 | *C4B* | complement C4B (Chido blood group) |
| P01031 | *C5* | complement C5 |
| P27708 | *CAD* | carbamoyl-phosphate synthetase 2, aspartate transcarbamylase, and dihydroorotase |
| O43852 | *CALU* | Calumenin |
| Q8IU85 | *CAMK1D* | calcium/calmodulin dependent protein kinase ID |
| Q9UQM7 | *CAMK2A* | calcium/calmodulin dependent protein kinase II alpha |
| Q13557 | *CAMK2D* | calcium/calmodulin dependent protein kinase II delta |
| Q14444 | *CAPRIN1* | cell cycle associated protein 1 |
| P47755 | *CAPZA2* | capping actin protein of muscle Z-line subunit alpha 2 |
| P47756 | *CAPZB* | capping actin protein of muscle Z-line subunit beta |
| Q8N163 | *CCAR2* | cell cycle and apoptosis regulator 2 |
| Q96NT0 | *CCDC115* | coiled-coil domain containing 115 |
| P0CW27 | *CCDC166* | coiled-coil domain containing 166 |
| P49368 | *CCT3* | chaperonin containing TCP1 subunit 3 |
| P50991 | *CCT4* | chaperonin containing TCP1 subunit 4 |
| P48643 | *CCT5* | chaperonin containing TCP1 subunit 5 |
| Q99832 | *CCT7* | chaperonin containing TCP1 subunit 7 |
| P50990 | *CCT8* | chaperonin containing TCP1 subunit 8 |
| P10966 | *CD8B* | CD8b molecule |
| P19022 | *CDH2* | cadherin 2 |
| Q96JP9 | *CDHR1* | cadherin related family member 1 |
| Q00535 | *CDK5* | cyclin dependent kinase 5 |
| Q96JB5 | *CDK5RAP3* | CDK5 regulatory subunit associated protein 3 |
| Q07065 | *CKAP4* | cytoskeleton associated protein 4 |
| Q14008 | *CKAP5* | cytoskeleton associated protein 5 |
| O75122 | *CLASP2* | cytoplasmic linker associated protein 2 |
| P51797 | *CLCN6* | chloride voltage-gated channel 6 |
| P09496 | *CLTA* | clathrin light chain A |
| Q5SYC1 | *CLVS2* | clavesin 2 |
| P09543 | *CNP* | 2',3'-cyclic nucleotide 3' phosphodiesterase |
| P53621 | *COPA* | COPI coat complex subunit alpha |
| O14579 | *COPE* | COPI coat complex subunit epsilon |
| Q9Y678 | *COPG1* | COPI coat complex subunit gamma 1 |
| Q96D53 | *COQ8B* | coenzyme Q8B |
| Q9UQ03 | *CORO2B* | coronin 2B |
| P00403 | *COX2* | cytochrome c oxidase subunit II |
| P13073 | *COX4I1* | cytochrome c oxidase subunit 4I1 |
| P20674 | *COX5A* | cytochrome c oxidase subunit 5A |
| P10606 | *COX5B* | cytochrome c oxidase subunit 5B |
| P09669 | *COX6C* | cytochrome c oxidase subunit 6C |
| P14406 | *COX7A2* | cytochrome c oxidase subunit 7A2 |
| O14548 | *COX7A2L* | cytochrome c oxidase subunit 7A2 like |
| P00450 | *CP* | Ceruloplasmin |
| P14384 | *CPM* | carboxypeptidase M |
| P15169 | *CPN1* | carboxypeptidase N subunit 1 |
| O95741 | *CPNE6* | copine 6 |
| Q9NQ79 | *CRTAC1* | cartilage acidic protein 1 |
| P02511 | *CRYAB* | crystallin alpha B |
| P56545 | *CTBP2* | C-terminal binding protein 2 |
| P35222 | *CTNNB1* | catenin beta 1 |
| O60716 | *CTNND1* | catenin delta 1 |
| Q93034 | *CUL5* | cullin 5 |
| Q02318 | *CYP27A1* | cytochrome P450 family 27 subfamily A member 1 |
| P14868 | *DARS1* | aspartyl-tRNA synthetase 1 |
| Q8WVC6 | *DCAKD* | dephospho-CoA kinase domain containing |
| O15075 | *DCLK1* | doublecortin like kinase 1 |
| Q8N568 | *DCLK2* | doublecortin like kinase 2 |
| Q16531 | *DDB1* | damage specific DNA binding protein 1 |
| Q92841 | *DDX17* | DEAD-box helicase 17 |
| O15523 | *DDX3Y* | DEAD-box helicase 3 Y-linked |
| P17844 | *DDX5* | DEAD-box helicase 5 |
| P17661 | *DES* | Desmin |
| P52429 | *DGKE* | diacylglycerol kinase epsilon |
| Q9Y485 | *DMXL1* | Dmx like 1 |
| P31689 | *DNAJA1* | DnaJ heat shock protein family (Hsp40) member A1 |
| Q96EY1 | *DNAJA3* | DnaJ heat shock protein family (Hsp40) member A3 |
| P25685 | *DNAJB1* | DnaJ heat shock protein family (Hsp40) member B1 |
| O00429 | *DNM1L* | dynamin 1 like |
| Q9C005 | *DPY30* | dpy-30 histone methyltransferase complex regulatory subunit |
| Q14195 | *DPYSL3* | dihydropyrimidinase like 3 |
| Q14204 | *DYNC1H1* | dynein cytoplasmic 1 heavy chain 1 |
| Q96EX3 | *DYNC2I2* | dynein 2 intermediate chain 2 |
| Q9NP97 | *DYNLRB1* | dynein light chain roadblock-type 1 |
| Q5JPH6 | *EARS2* | glutamyl-tRNA synthetase 2, mitochondrial |
| P68104 | *EEF1A1* | eukaryotic translation elongation factor 1 alpha 1 |
| Q05639 | *EEF1A2* | eukaryotic translation elongation factor 1 alpha 2 |
| P29692 | *EEF1D* | eukaryotic translation elongation factor 1 delta |
| P26641 | *EEF1G* | eukaryotic translation elongation factor 1 gamma |
| P13639 | *EEF2* | eukaryotic translation elongation factor 2 |
| P60842 | *EIF4A1* | eukaryotic translation initiation factor 4A1 |
| Q8N336 | *ELMOD1* | ELMO domain containing 1 |
| Q15369 | *ELOC* | elongin C |
| P50402 | *EMD* | Emerin |
| Q9Y2J2 | *EPB41L3* | erythrocyte membrane protein band 4.1 like 3 |
| Q7L775 | *EPM2AIP1* | EPM2A interacting protein 1 |
| O75477 | *ERLIN1* | ER lipid raft associated 1 |
| O94905 | *ERLIN2* | ER lipid raft associated 2 |
| B1AK53 | *ESPN* | Espin |
| Q9BSJ8 | *ESYT1* | extended synaptotagmin 1 |
| P00734 | *F2* | coagulation factor II, thrombin |
| P12259 | *F5* | coagulation factor V |
| Q96CS3 | *FAF2* | Fas associated factor family member 2 |
| Q9NZB2 | *FAM120A* | family with sequence similarity 120A |
| Q96EK7 | *FAM120B* | family with sequence similarity 120B |
| P23142 | *FBLN1* | fibulin 1 |
| Q9UBX5 | *FBLN5* | fibulin 5 |
| Q9UK22 | *FBXO2* | F-box protein 2 |
| Q9NVF7 | *FBXO28* | F-box protein 28 |
| Q86UX7 | *FERMT3* | fermitin family member 3 |
| Q14318 | *FKBP8* | FKBP prolyl isomerase 8 |
| Q13045 | *FLII* | FLII actin remodeling protein |
| Q14254 | *FLOT2* | flotillin 2 |
| Q9H479 | *FN3K* | fructosamine 3 kinase |
| Q96CU9 | *FOXRED1* | FAD dependent oxidoreductase domain containing 1 |
| Q96I24 | *FUBP3* | far upstream element binding protein 3 |
| P51114 | *FXR1* | FMR1 autosomal homolog 1 |
| Q13283 | *G3BP1* | G3BP stress granule assembly factor 1 |
| O14976 | *GAK* | cyclin G associated kinase |
| Q969S9 | *GFM2* | GTP dependent ribosome recycling factor mitochondrial 2 |
| O94925 | *GLS* | Glutaminase |
| P29992 | *GNA11* | G protein subunit alpha 11 |
| P04899 | *GNAI2* | G protein subunit alpha i2 |
| Q5JWF2 | *GNAS* | GNAS complex locus |
| P19086 | *GNAZ* | G protein subunit alpha z |
| Q08379 | *GOLGA2* | golgin A2 |
| Q08378 | *GOLGA3* | golgin A3 |
| O75715 | *GPX5* | glutathione peroxidase 5 |
| Q4V328 | *GRIPAP1* | GRIP1 associated protein 1 |
| P06396 | *GSN* | Gelsolin |
| Q8N442 | *GUF1* | GUF1 homolog, GTPase |
| P13807 | *GYS1* | glycogen synthase 1 |
| Q92522 | *H1-10* | H1.10 linker histone |
| P40939 | *HADHA* | hydroxyacyl-CoA dehydrogenase trifunctional multienzyme complex subunit alpha |
| P55084 | *HADHB* | hydroxyacyl-CoA dehydrogenase trifunctional multienzyme complex subunit beta |
| O60741 | *HCN1* | hyperpolarization activated cyclic nucleotide gated potassium channel 1 |
| Q00341 | *HDLBP* | high density lipoprotein binding protein |
| P52789 | *HK2* | hexokinase 2 |
| Q2TB90 | *HKDC1* | hexokinase domain containing 1 |
| P01892 | *HLA-A* | HLA class I histocompatibility antigen, A-2 alpha chain |
| P01889 | *HLA-B* | major histocompatibility complex, class I, B |
| P31943 | *HNRNPH1* | heterogeneous nuclear ribonucleoprotein H1 |
| Q9BUJ2 | *HNRNPUL1* | heterogeneous nuclear ribonucleoprotein U like 1 |
| Q53GQ0 | *HSD17B12* | hydroxysteroid 17-beta dehydrogenase 12 |
| O43301 | *HSPA12A* | heat shock protein family A (Hsp70) member 12A |
| P54652 | *HSPA2* | heat shock protein family A (Hsp70) member 2 |
| P11142 | *HSPA8* | heat shock protein family A (Hsp70) member 8 |
| P10809 | *HSPD1* | heat shock protein family D (Hsp60) member 1 |
| Q9Y4L1 | *HYOU1* | hypoxia up-regulated 1 |
| Q9NSE4 | *IARS2* | isoleucyl-tRNA synthetase 2, mitochondrial |
| O43837 | *IDH3B* | isocitrate dehydrogenase (NAD(+)) 3 non-catalytic subunit beta |
| Q96ID5 | *IGSF21* | immunoglobin superfamily member 21 |
| A1L0T0 | *ILVBL* | ilvB acetolactate synthase like |
| Q16891 | *IMMT* | inner membrane mitochondrial protein |
| P20839 | *IMPDH1* | inosine monophosphate dehydrogenase 1 |
| Q16352 | *INA* | internexin neuronal intermediate filament protein alpha |
| Q6DN90 | *IQSEC1* | IQ motif and Sec7 domain ArfGEF 1 |
| Q06033 | *ITIH3* | inter-alpha-trypsin inhibitor heavy chain 3 |
| Q9Y287 | *ITM2B* | integral membrane protein 2B |
| Q9NQX7 | *ITM2C* | integral membrane protein 2C |
| Q14721 | *KCNB1* | potassium voltage-gated channel subfamily B member 1 |
| Q6ZWB6 | *KCTD8* | potassium channel tetramerization domain containing 8 |
| Q9ULH0 | *KIDINS220* | kinase D interacting substrate 220 |
| Q7Z4S6 | *KIF21A* | kinesin family member 21A |
| O14686 | *KMT2D* | lysine methyltransferase 2D |
| P32004 | *L1CAM* | L1 cell adhesion molecule |
| A8MY62 | *LACTBL1* | Putative beta-lactamase-like 1 |
| Q9P2J5 | *LARS1* | leucyl-tRNA synthetase 1 |
| Q9BXB1 | *LGR4* | leucine rich repeat containing G protein-coupled receptor 4 |
| P49916 | *LIG3* | DNA ligase 3 |
| P49257 | *LMAN1* | lectin, mannose binding 1 |
| Q07954 | *LRP1* | LDL receptor related protein 1 |
| Q13449 | *LSAMP* | limbic system associated membrane protein |
| Q3MHD2 | *LSM12* | LSM12 homolog |
| O95372 | *LYPLA2* | lysophospholipase 2 |
| Q8WXG6 | *MADD* | MAP kinase activating death domain |
| P46821 | *MAP1B* | microtubule associated protein 1B |
| Q96JE9 | *MAP6* | microtubule associated protein 6 |
| Q14244 | *MAP7* | microtubule associated protein 7 |
| Q15555 | *MAPRE2* | microtubule associated protein RP/EB family member 2 |
| P56192 | *MARS1* | methionyl-tRNA synthetase 1 |
| Q96N66 | *MBOAT7* | membrane bound O-acyltransferase domain containing 7 |
| C9JLW8 | *MCRIP1* | MAPK regulated corepressor interacting protein 1 |
| P55083 | *MFAP4* | microfibril associated protein 4 |
| Q9GZY8 | *MFF* | mitochondrial fission factor |
| Q15773 | *MLF2* | myeloid leukemia factor 2 |
| Q13015 | *MLLT11* | MLLT11 transcription factor 7 cofactor |
| Q13724 | *MOGS* | mannosyl-oligosaccharide glucosidase |
| Q14168 | *MPP2* | membrane palmitoylated protein 2 |
| O43347 | *MSI1* | musashi RNA binding protein 1 |
| Q96DH6 | *MSI2* | musashi RNA binding protein 2 |
| P11586 | *MTHFD1* | methylenetetrahydrofolate dehydrogenase, cyclohydrolase and formyltetrahydrofolate synthetase 1 |
| O75592 | *MYCBP2* | MYC binding protein 2 |
| P35580 | *MYH10* | myosin heavy chain 10 |
| O14950 | *MYL12A* | myosin light chain 12A |
| P12829 | *MYL4* | myosin light chain 4 |
| P60660 | *MYL6* | myosin light chain 6 |
| P24844 | *MYL9* | myosin light chain 9 |
| Q92614 | *MYO18A* | myosin XVIIIA |
| O00159 | *MYO1C* | myosin IC |
| Q9Y4I1 | *MYO5A* | myosin VA |
| Q9ULV0 | *MYO5B* | myosin VB |
| Q13402 | *MYO7A* | myosin VIIA |
| P43490 | *NAMPT* | nicotinamide phosphoribosyltransferase |
| P13591 | *NCAM1* | neural cell adhesion molecule 1 |
| P19338 | *NCL* | Nucleolin |
| O95299 | *NDUFA10* | NADH:ubiquinone oxidoreductase subunit A10 |
| Q9UI09 | *NDUFA12* | NADH:ubiquinone oxidoreductase subunit A12 |
| Q9P0J0 | *NDUFA13* | NADH:ubiquinone oxidoreductase subunit A13 |
| O43678 | *NDUFA2* | NADH:ubiquinone oxidoreductase subunit A2 |
| O95167 | *NDUFA3* | NADH:ubiquinone oxidoreductase subunit A3 |
| Q16718 | *NDUFA5* | NADH:ubiquinone oxidoreductase subunit A5 |
| P56556 | *NDUFA6* | NADH:ubiquinone oxidoreductase subunit A6 |
| O95182 | *NDUFA7* | NADH:ubiquinone oxidoreductase subunit A7 |
| Q16795 | *NDUFA9* | NADH:ubiquinone oxidoreductase subunit A9 |
| O96000 | *NDUFB10* | NADH:ubiquinone oxidoreductase subunit B10 |
| O95168 | *NDUFB4* | NADH:ubiquinone oxidoreductase subunit B4 |
| O43674 | *NDUFB5* | NADH:ubiquinone oxidoreductase subunit B5 |
| O95139 | *NDUFB6* | NADH:ubiquinone oxidoreductase subunit B6 |
| O95169 | *NDUFB8* | NADH:ubiquinone oxidoreductase subunit B8 |
| Q9Y6M9 | *NDUFB9* | NADH:ubiquinone oxidoreductase subunit B9 |
| P28331 | *NDUFS1* | NADH:ubiquinone oxidoreductase core subunit S1 |
| O75306 | *NDUFS2* | NADH:ubiquinone oxidoreductase core subunit S2 |
| O75489 | *NDUFS3* | NADH:ubiquinone oxidoreductase core subunit S3 |
| O43181 | *NDUFS4* | NADH:ubiquinone oxidoreductase subunit S4 |
| O43920 | *NDUFS5* | NADH:ubiquinone oxidoreductase subunit S5 |
| O75380 | *NDUFS6* | NADH:ubiquinone oxidoreductase subunit S6 |
| O75251 | *NDUFS7* | NADH:ubiquinone oxidoreductase core subunit S7 |
| O00217 | *NDUFS8* | NADH:ubiquinone oxidoreductase core subunit S8 |
| P49821 | *NDUFV1* | NADH:ubiquinone oxidoreductase core subunit V1 |
| P19404 | *NDUFV2* | NADH:ubiquinone oxidoreductase core subunit V2 |
| P56181 | *NDUFV3* | NADH:ubiquinone oxidoreductase subunit V3 |
| P12036 | *NEFH* | neurofilament heavy |
| P07196 | *NEFL* | neurofilament light |
| P07197 | *NEFM* | neurofilament medium |
| P08651 | *NFIC* | nuclear factor I C |
| Q9Y2X3 | *NOP58* | NOP58 ribonucleoprotein |
| P46459 | *NSF* | N-ethylmaleimide sensitive factor, vesicle fusing ATPase |
| Q8TB37 | *NUBPL* | nucleotide binding protein like |
| Q9BRJ7 | *NUDT16L1* | nudix hydrolase 16 like 1 |
| P04181 | *OAT* | ornithine aminotransferase |
| Q14982 | *OPCML* | opioid binding protein/cell adhesion molecule like |
| Q9BZF1 | *OSBPL8* | oxysterol binding protein like 8 |
| Q9UQ80 | *PA2G4* | proliferation-associated 2G4 |
| P11940 | *PABPC1* | poly(A) binding protein cytoplasmic 1 |
| Q13310 | *PABPC4* | poly(A) binding protein cytoplasmic 4 |
| Q6VY07 | *PACS1* | phosphofurin acidic cluster sorting protein 1 |
| Q9Y2J8 | *PADI2* | peptidyl arginine deiminase 2 |
| O75781 | *PALM* | Paralemmin |
| Q96AQ6 | *PBXIP1* | PBX homeobox interacting protein 1 |
| P11498 | *PC* | pyruvate carboxylase |
| Q15365 | *PCBP1* | poly(rC) binding protein 1 |
| O95206 | *PCDH8* | protocadherin 8 |
| Q9Y5F8 | *PCDHGB7* | protocadherin gamma subfamily B, 7 |
| P20941 | *PDC* | Phosducin |
| P16499 | *PDE6A* | phosphodiesterase 6A |
| P11177 | *PDHB* | pyruvate dehydrogenase E1 subunit beta |
| P17858 | *PFKL* | phosphofructokinase, liver type |
| Q01813 | *PFKP* | phosphofructokinase, platelet |
| P07737 | *PFN1* | profilin 1 |
| O00264 | *PGRMC1* | progesterone receptor membrane component 1 |
| P42356 | *PI4KA* | phosphatidylinositol 4-kinase alpha |
| P78356 | *PIP4K2B* | phosphatidylinositol-5-phosphate 4-kinase type 2 beta |
| Q99959 | *PKP2* | plakophilin 2 |
| Q99569 | *PKP4* | plakophilin 4 |
| Q15149 | *PLEC* | Plectin |
| P24928 | *POLR2A* | RNA polymerase II subunit A |
| P50336 | *PPOX* | protoporphyrinogen oxidase |
| Q6ZSY5 | *PPP1R3F* | protein phosphatase 1 regulatory subunit 3F |
| P62714 | *PPP2CB* | protein phosphatase 2 catalytic subunit beta |
| Q66LE6 | *PPP2R2D* | protein phosphatase 2 regulatory subunit Bdelta |
| Q9HCU5 | *PREB* | prolactin regulatory element binding |
| P51888 | *PRELP* | proline and arginine rich end leucine rich repeat protein |
| P22694 | *PRKACB* | protein kinase cAMP-activated catalytic subunit beta |
| P60891 | *PRPS1* | phosphoribosyl pyrophosphate synthetase 1 |
| P28066 | *PSMA5* | proteasome 20S subunit alpha 5 |
| P62191 | *PSMC1* | proteasome 26S subunit, ATPase 1 |
| P35998 | *PSMC2* | proteasome 26S subunit, ATPase 2 |
| P17980 | *PSMC3* | proteasome 26S subunit, ATPase 3 |
| P62333 | *PSMC6* | proteasome 26S subunit, ATPase 6 |
| Q99460 | *PSMD1* | proteasome 26S subunit, non-ATPase 1 |
| P10586 | *PTPRF* | protein tyrosine phosphatase receptor type F |
| Q13332 | *PTPRS* | protein tyrosine phosphatase receptor type S |
| Q96C36 | *PYCR2* | pyrroline-5-carboxylate reductase 2 |
| P11216 | *PYGB* | glycogen phosphorylase B |
| P47897 | *QARS1* | glutaminyl-tRNA synthetase 1 |
| P63244 | *RACK1* | receptor for activated C kinase 1 |
| P62826 | *RAN* | RAN, member RAS oncogene family |
| P54136 | *RARS1* | arginyl-tRNA synthetase 1 |
| Q15291 | *RBBP5* | RB binding protein 5, histone lysine methyltransferase complex subunit |
| Q15293 | *RCN1* | reticulocalbin 1 |
| Q14257 | *RCN2* | reticulocalbin 2 |
| Q96NR8 | *RDH12* | retinol dehydrogenase 12 |
| Q8IXI1 | *RHOT2* | ras homolog family member T2 |
| Q6R327 | *RICTOR* | RPTOR independent companion of MTOR complex 2 |
| P62906 | *RPL10A* | ribosomal protein L10a |
| P30050 | *RPL12* | ribosomal protein L12 |
| P40429 | *RPL13A* | ribosomal protein L13a |
| P18621 | *RPL17* | ribosomal protein L17 |
| Q07020 | *RPL18* | ribosomal protein L18 |
| P84098 | *RPL19* | ribosomal protein L19 |
| P62829 | *RPL23* | ribosomal protein L23 |
| P61254 | *RPL26* | ribosomal protein L26 |
| P61353 | *RPL27* | ribosomal protein L27 |
| P46776 | *RPL27A* | ribosomal protein L27a |
| P62888 | *RPL30* | ribosomal protein L30 |
| P36578 | *RPL4* | ribosomal protein L4 |
| P62424 | *RPL7A* | ribosomal protein L7a |
| P05388 | *RPLP0* | ribosomal protein lateral stalk subunit P0 |
| P04843 | *RPN1* | ribophorin I |
| P62277 | *RPS13* | ribosomal protein S13 |
| P62263 | *RPS14* | ribosomal protein S14 |
| P62249 | *RPS16* | ribosomal protein S16 |
| P62269 | *RPS18* | ribosomal protein S18 |
| P39019 | *RPS19* | ribosomal protein S19 |
| P15880 | *RPS2* | ribosomal protein S2 |
| P60866 | *RPS20* | ribosomal protein S20 |
| P62847 | *RPS24* | ribosomal protein S24 |
| P62979 | *RPS27A* | ribosomal protein S27a |
| P62701 | *RPS4X* | ribosomal protein S4 X-linked |
| Q8TD47 | *RPS4Y2* | ribosomal protein S4 Y-linked 2 |
| P62241 | *RPS8* | ribosomal protein S8 |
| P08865 | *RPSA* | ribosomal protein SA |
| O00442 | *RTCA* | RNA 3'-terminal phosphate cyclase |
| Q9Y3I0 | *RTCB* | RNA 2',3'-cyclic phosphate and 5'-OH ligase |
| Q7L099 | *RUFY3* | RUN and FYVE domain containing 3 |
| Q9Y265 | *RUVBL1* | RuvB like AAA ATPase 1 |
| Q01118 | *SCN7A* | sodium voltage-gated channel alpha subunit 7 |
| P31040 | *SDHA* | succinate dehydrogenase complex flavoprotein subunit A |
| P55735 | *SEC13* | SEC13 homolog, nuclear pore and COPII coat complex component |
| O15027 | *SEC16A* | SEC16 homolog A, endoplasmic reticulum export factor |
| O75396 | *SEC22B* | SEC22 homolog B, vesicle trafficking protein |
| Q15393 | *SF3B3* | splicing factor 3b subunit 3 |
| Q9NR46 | *SH3GLB2* | SH3 domain containing GRB2 like, endophilin B2 |
| P55011 | *SLC12A2* | solute carrier family 12 member 2 |
| Q9H2X9 | *SLC12A5* | solute carrier family 12 member 5 |
| P53985 | *SLC16A1* | solute carrier family 16 member 1 |
| Q02978 | *SLC25A11* | solute carrier family 25 member 11 |
| O75746 | *SLC25A12* | solute carrier family 25 member 12 |
| Q00325 | *SLC25A3* | solute carrier family 25 member 3 |
| P12236 | *SLC25A6* | solute carrier family 25 member 6 |
| Q9Y2P4 | *SLC27A6* | solute carrier family 27 member 6 |
| P11166 | *SLC2A1* | solute carrier family 2 member 1 |
| Q6U841 | *SLC4A10* | solute carrier family 4 member 10 |
| Q2Y0W8 | *SLC4A8* | solute carrier family 4 member 8 |
| Q13884 | *SNTB1* | syntrophin beta 1 |
| Q13425 | *SNTB2* | syntrophin beta 2 |
| Q96PQ0 | *SORCS2* | sortilin related VPS10 domain containing receptor 2 |
| Q07889 | *SOS1* | SOS Ras/Rac guanine nucleotide exchange factor 1 |
| Q01082 | *SPTBN1* | spectrin beta, non-erythrocytic 1 |
| Q9C0H9 | *SRCIN1* | SRC kinase signaling inhibitor 1 |
| P19623 | *SRM* | spermidine synthase |
| P37108 | *SRP14* | signal recognition particle 14 |
| P49458 | *SRP9* | signal recognition particle 9 |
| Q9UJZ1 | *STOML2* | stomatin like 2 |
| Q9UNE7 | *STUB1* | STIP1 homology and U-box containing protein 1 |
| P61764 | *STXBP1* | syntaxin binding protein 1 |
| Q9P2R7 | *SUCLA2* | succinate-CoA ligase ADP-forming subunit beta |
| P53597 | *SUCLG1* | succinate-CoA ligase GDP/ADP-forming subunit alpha |
| Q8IX01 | *SUGP2* | SURP and G-patch domain containing 2 |
| O15260 | *SURF4* | surfeit 4 |
| O60506 | *SYNCRIP* | synaptotagmin binding cytoplasmic RNA interacting protein |
| O15061 | *SYNM* | synemin |
| Q9BT88 | *SYT11* | synaptotagmin 11 |
| P26639 | *TARS1* | threonyl-tRNA synthetase 1 |
| A2RTX5 | *TARS3* | threonyl-tRNA synthetase 3 |
| P17987 | *TCP1* | t-complex 1 |
| Q9NZ01 | *TECR* | trans-2,3-enoyl-CoA reductase |
| Q8IYQ7 | *THNSL1* | threonine synthase like 1 |
| Q9UKI8 | *TLK1* | tousled like kinase 1 |
| P49755 | *TMED10* | transmembrane p24 trafficking protein 10 |
| Q24JP5 | *TMEM132A* | transmembrane protein 132A |
| Q8N511 | *TMEM199* | transmembrane protein 199 |
| Q96Q45 | *TMEM237* | transmembrane protein 237 |
| Q9NZR1 | *TMOD2* | tropomodulin 2 |
| Q92973 | *TNPO1* | transportin 1 |
| O60784 | *TOM1* | target of myb1 membrane trafficking protein |
| Q6ZVM7 | *TOM1L2* | target of myb1 like 2 membrane trafficking protein |
| Q9Y5R8 | *TRAPPC1* | trafficking protein particle complex 1 |
| Q9UL33 | *TRAPPC2L* | trafficking protein particle complex 2 like |
| P14373 | *TRIM27* | tripartite motif containing 27 |
| Q71U36 | *TUBA1A* | tubulin alpha 1a |
| P68366 | *TUBA4A* | tubulin alpha 4a |
| A6NHL2 | *TUBAL3* | tubulin alpha like 3 |
| P07437 | *TUBB* | tubulin beta class I |
| Q13885 | *TUBB2A* | tubulin beta 2A class IIa |
| Q9BVA1 | *TUBB2B* | tubulin beta 2B class IIb |
| Q13509 | *TUBB3* | tubulin beta 3 class III |
| P04350 | *TUBB4A* | tubulin beta 4A class IVa |
| P68371 | *TUBB4B* | tubulin beta 4B class IVb |
| P23258 | *TUBG1* | tubulin gamma 1 |
| P49411 | *TUFM* | Tu translation elongation factor, mitochondrial |
| O00294 | *TULP1* | TUB like protein 1 |
| Q14157 | *UBAP2L* | ubiquitin associated protein 2 like |
| Q92900 | *UPF1* | UPF1 RNA helicase and ATPase |
| P31930 | *UQCRC1* | ubiquinol-cytochrome c reductase core protein 1 |
| O14949 | *UQCRQ* | ubiquinol-cytochrome c reductase complex III subunit VII |
| P45974 | *USP5* | ubiquitin specific peptidase 5 |
| Q93008 | *USP9X* | ubiquitin specific peptidase 9 X-linked |
| Q08AM6 | *VAC14* | VAC14 component of PIKFYVE complex |
| O95292 | *VAPB* | VAMP associated protein B and C |
| P26640 | *VARS1* | valyl-tRNA synthetase 1 |
| P45880 | *VDAC2* | voltage dependent anion channel 2 |
| P08670 | *VIM* | vimentin |
| Q9H269 | *VPS16* | VPS16 core subunit of CORVET and HOPS complexes |
| Q9NP79 | *VTA1* | vesicle trafficking 1 |
| A3KMH1 | *VWA8* | von Willebrand factor A domain containing 8 |
| O94967 | *WDR47* | WD repeat domain 47 |
| Q9Y4E6 | *WDR7* | WD repeat domain 7 |
| P27348 | *YWHAQ* | tyrosine 3-monooxygenase/tryptophan 5-monooxygenase activation protein theta |

**Supplementary Table S2:** Proteins identified in this study and highly enriched in the retina (data obtained from the Human Atlas Protein (available online: www.proteinatlas.org)).

| **Uniprot accession** | **Gene name** | **Protein name** | **Expression in the retina** |
| --- | --- | --- | --- |
| O60741 | *HCN1* | Potassium/sodium hyperpolarization-activated cyclic nucleotide-gated channel 1 | Photoreceptors |
| Q14721 | *KCNB1* | Potassium voltage-gated channel subfamily B member 1 | Photoreceptors |
| Q96JP9 | *CDHR1* | Cadherin-related family member | Photoreceptors |
| Q9NQ79 | *CRTAC1* | Cartilage acidic protein | N.A |
| O43347 | *MSI1* | RNA-binding protein Musashi homolog 1 | Photoreceptors |
| O00294 | *TULP1* | Tubby-related protein 1 | Photoreceptors |
| Q13402 | *MYO7A* | Unconventional myosin-VIIa | Photoreceptors |
| P20941 | *PDC* | Phosducin | Photoreceptors |
| P16499 | *PDE6A* | Rod cGMP-specific 3',5'-cyclic phosphodiesterase subunit alpha | Photoreceptors |
|  |  |  |  |

**Supplementary Table S3.** GO-Terms enrichment analysis of the 9 proteins identified in our study and highly enriched in the retinal tissue (Supplementary Table S2). Analysis obtained using DAVID online database (version 6.8). Enriched categories identified are those with p-value < 0.05.

| **GO-Term** | **p-value** | **Number of proteins** | **Associated proteins** |
| --- | --- | --- | --- |
| Visual perception | 1.4 E -4 | 4 | MYO7A ,PDC, PDE6A, TULP1 |
| Eye photoreceptor cell development | 3.7E-2 | 2 | MYO7A, TULP1 |


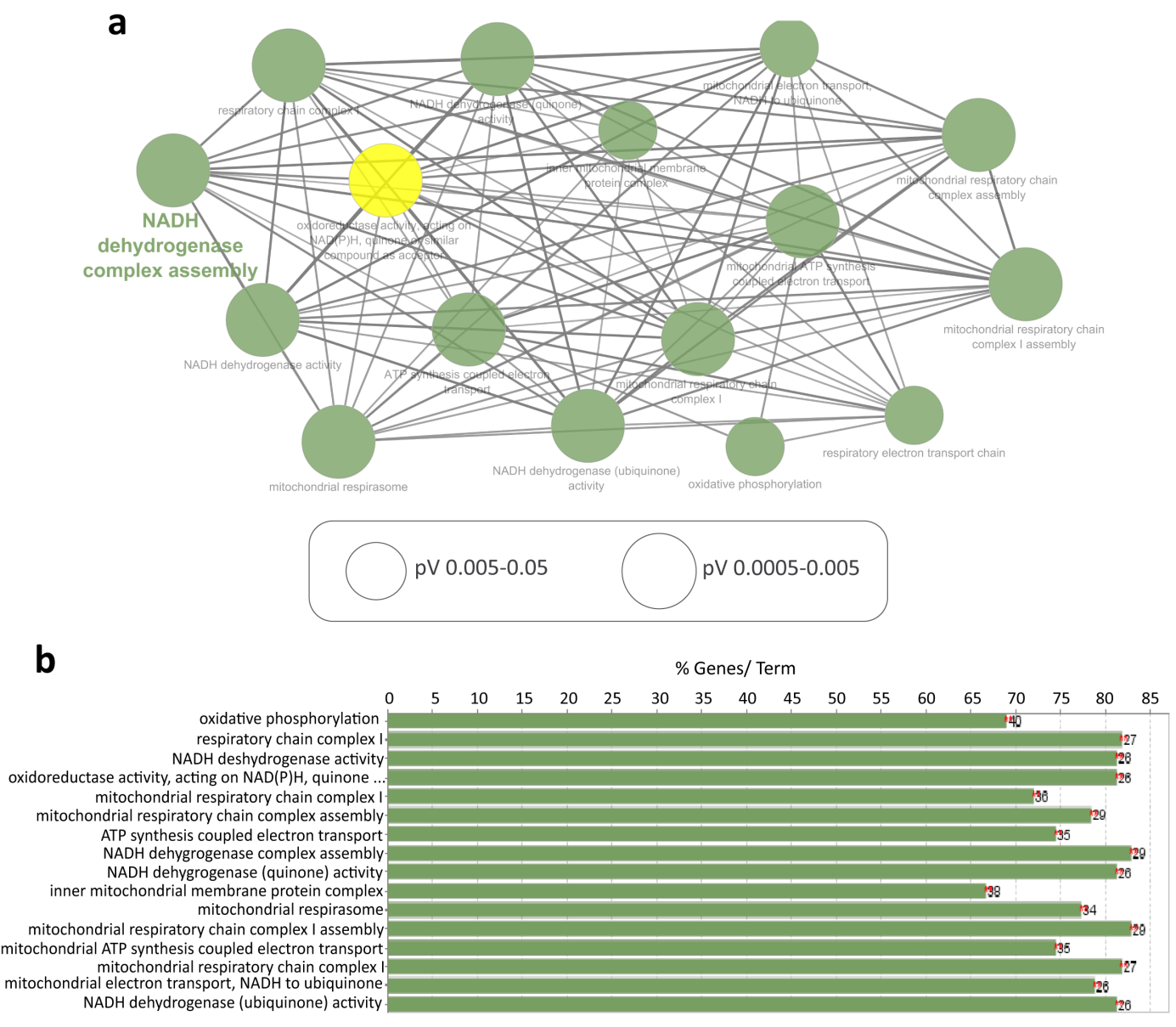


Supplementary Figure S2. ITM2B interactome in the retina. Ontology term enrichment and visualization were obtained using the ClueGo plug-in (Version 2.5.6) within the Cytoscape software (version 3.7.1) (a) GO-term network analysis (b) Functional clusters and pathways identified with enriched proteins . %Genes/Term corresponds to the proportion of genes enriched in the functional clusters. Bars with the same color belong to the same functional cluster.

**Supplementary Table S4.** GO-Terms enrichment analysis of the ITM2B retinal interactome (457 proteins).

| **GO-Term** | **% Associated Genes** | **Nr Genes** | **Associated Proteins** |
| --- | --- | --- | --- |
| oxidative phosphorylation | 68,97 | 40,00 | ATP5F1A, ATP5F1B, ATP5F1C, ATP5PF, COX2, COX4I1, COX5A, COX5B, COX6C, COX7A2L, NDUFA10, NDUFA12, NDUFA13, NDUFA2, NDUFA3, NDUFA5, NDUFA6, NDUFA7, NDUFA9, NDUFB10, NDUFB4, NDUFB5, NDUFB6, NDUFB8, NDUFB9, NDUFS1, NDUFS2, NDUFS3, NDUFS4, NDUFS5, NDUFS6, NDUFS7, NDUFS8, NDUFV1, NDUFV2, NDUFV3, SDHA, STOML2, UQCRC1, UQCRQ |
| respiratory chain complex I | 81,82 | 27,00 | FOXRED1, NDUFA10, NDUFA12, NDUFA13, NDUFA2, NDUFA3, NDUFA5, NDUFA6, NDUFA7, NDUFA9, NDUFB10, NDUFB4, NDUFB5, NDUFB6, NDUFB8, NDUFB9, NDUFS1, NDUFS2, NDUFS3, NDUFS4, NDUFS5, NDUFS6, NDUFS7, NDUFS8, NDUFV1, NDUFV2, NDUFV3 |
| NADH dehydrogenase activity | 81,25 | 26,00 | NDUFA10, NDUFA12, NDUFA13, NDUFA2, NDUFA3, NDUFA5, NDUFA6, NDUFA7, NDUFA9, NDUFB10, NDUFB4, NDUFB5, NDUFB6, NDUFB8, NDUFB9, NDUFS1, NDUFS2, NDUFS3, |
| oxidoreductase activity, acting on NAD(P)H, quinone or similar compound as acceptor | 81,25 | 26,00 | NDUFA10, NDUFA12, NDUFA13, NDUFA2, NDUFA3, NDUFA5, NDUFA6, NDUFA7, NDUFA9, NDUFB10, NDUFB4, NDUFB5, NDUFB6, NDUFB8, NDUFB9, NDUFS1, NDUFS2, NDUFS3, |
| respiratory electron transport chain | 72,00 | 36,00 | COX2, COX4I1, COX5A, COX5B, COX6C, COX7A2L, NDUFA10, NDUFA12, NDUFA13, NDUFA2, NDUFA3, NDUFA5, NDUFA6, NDUFA7, NDUFA9, NDUFB10, NDUFB4, NDUFB5, |
| mitochondrial respiratory chain complex assembly | 78,38 | 29,00 | ACAD9, FOXRED1, NDUFA10, NDUFA12, NDUFA13, NDUFA2, NDUFA3, NDUFA5, NDUFA6, NDUFA7, NDUFA9, NDUFB10, NDUFB4, NDUFB5, NDUFB6, NDUFB8, NDUFB9, NDUFS1, NDUFS2, NDUFS3, NDUFS4, NDUFS5, NDUFS6, NDUFS7, NDUFS8, NDUFV1, NDUFV2, NDUFV3, NUBPL |
| ATP synthesis coupled electron transport | 74,47 | 35,00 | COX2, COX4I1, COX5A, COX5B, COX6C, COX7A2L, NDUFA10, NDUFA12, NDUFA13, NDUFA2, NDUFA3, NDUFA5, NDUFA6, NDUFA7, NDUFA9, NDUFB10, NDUFB4, NDUFB5, NDUFB6, NDUFB8, NDUFB9, NDUFS1, NDUFS2, NDUFS3, NDUFS4, NDUFS5, NDUFS6, NDUFS7, NDUFS8, NDUFV1, NDUFV2, NDUFV3, SDHA, UQCRC1, UQCRQ |
| NADH dehydrogenase complex assembly | 82,86 | 29,00 | ACAD9, FOXRED1, NDUFA10, NDUFA12, NDUFA13, NDUFA2, NDUFA3, NDUFA5, NDUFA6, NDUFA7, NDUFA9, NDUFB10, NDUFB4, NDUFB5, NDUFB6, NDUFB8, NDUFB9, NDUFS1, |
| NADH dehydrogenase (quinone) activity | 81,25 | 26,00 | NDUFA10, NDUFA12, NDUFA13, NDUFA2, NDUFA3, NDUFA5, NDUFA6, NDUFA7, NDUFA9, NDUFB10, NDUFB4, NDUFB5, NDUFB6, NDUFB8, NDUFB9, NDUFS1, NDUFS2, NDUFS3, |
| inner mitochondrial membrane protein complex | 66,67 | 38,00 | ATP5F1A, ATP5F1B, ATP5F1C, ATP5PF, COX4I1, COX5A, FOXRED1, IMMT, NDUFA10, NDUFA12, NDUFA13, NDUFA2, NDUFA3, NDUFA5, NDUFA6, NDUFA7, NDUFA9, NDUFB10, NDUFB4, NDUFB5, NDUFB6, NDUFB8, NDUFB9, NDUFS1, NDUFS2, NDUFS3, NDUFS4, NDUFS5, NDUFS6, NDUFS7, NDUFS8, NDUFV1, NDUFV2, NDUFV3, SDHA, SLC25A6, UQCRC1, UQCRQ] |
| mitochondrial respirasome | 77,27 | 34,00 | COX4I1, COX5A, COX7A2, COX7A2L, FOXRED1, NDUFA10, NDUFA12, NDUFA13, NDUFA2, NDUFA3, NDUFA5, NDUFA6, NDUFA7, NDUFA9, NDUFB10, NDUFB4, NDUFB5, NDUFB6, |
| mitochondrial respiratory chain complex I assembly | 82,86 | 29,00 | ACAD9, FOXRED1, NDUFA10, NDUFA12, NDUFA13, NDUFA2, NDUFA3, NDUFA5, NDUFA6, NDUFA7, NDUFA9, NDUFB10, NDUFB4, NDUFB5, NDUFB6, NDUFB8, NDUFB9, NDUFS1, |
| mitochondrial ATP synthesis coupled electron transport | 74,47 | 35,00 | COX2, COX4I1, COX5A, COX5B, COX6C, COX7A2L, NDUFA10, NDUFA12, NDUFA13, NDUFA2, NDUFA3, NDUFA5, NDUFA6, NDUFA7, NDUFA9, NDUFB10, NDUFB4, NDUFB5, NDUFB6, NDUFB8, NDUFB9, NDUFS1, NDUFS2, NDUFS3, NDUFS4, NDUFS5, NDUFS6, NDUFS7, NDUFS8, NDUFV1, NDUFV2, NDUFV3, SDHA, UQCRC1, UQCRQ |
| mitochondrial respiratory chain complex I | 81,82 | 27,00 | FOXRED1, NDUFA10, NDUFA12, NDUFA13, NDUFA2, NDUFA3, NDUFA5, NDUFA6, NDUFA7, NDUFA9, NDUFB10, NDUFB4, NDUFB5, NDUFB6, NDUFB8, NDUFB9, NDUFS1, NDUFS2, NDUFS3, NDUFS4, NDUFS5, NDUFS6, NDUFS7, NDUFS8, NDUFV1, NDUFV2, NDUFV3 |
| mitochondrial electron transport, NADH to ubiquinone | 78,79 | 26,00 | NDUFA10, NDUFA12, NDUFA13, NDUFA2, NDUFA3, NDUFA5, NDUFA6, NDUFA7, NDUFA9, NDUFB10, NDUFB4, NDUFB5, NDUFB6, NDUFB8, NDUFB9, NDUFS1, NDUFS2, NDUFS3, NDUFS4, NDUFS5, NDUFS6, NDUFS7, NDUFS8, NDUFV1, NDUFV2, NDUFV3 |
| NADH dehydrogenase (ubiquinone) activity | 81,25 | 26,00 | NDUFA10, NDUFA12, NDUFA13, NDUFA2, NDUFA3, NDUFA5, NDUFA6, NDUFA7, NDUFA9, NDUFB10, NDUFB4, NDUFB5, NDUFB6, NDUFB8, NDUFB9, NDUFS1, NDUFS2, NDUFS3, NDUFS4, NDUFS5, NDUFS6, NDUFS7, NDUFS8, NDUFV1, NDUFV2, NDUFV3 |

Supplementary Table S5. Curated list representing 140 proteins identified by LC-MS/MS and purified with the mouse and rabbit anti-ITM2B antibodies (FC >2 and p-value<0.01).

| **Uniprot accession** | **Gene name** | **Protein name** |
| --- | --- | --- |
| P01023 | *A2M* | alpha-2-macroglobulin |
| P61221 | *ABCE1* | ATP binding cassette subfamily E member 1 |
| P24666 | *ACP1* | acid phosphatase 1 |
| P60709 | *ACTB* | actin beta |
| P68032 | *ACTC1* | actin alpha cardiac muscle 1 |
| P61160 | *ACTR2* | actin related protein 2 |
| P02768 | *ALB* | albumin |
| O95782 | *AP2A1* | adaptor related protein complex 2 subunit alpha 1 |
| Q8N6H7 | *ARFGAP2* | ADP ribosylation factor GTPase activating protein 2 |
| P40616 | *ARL1* | ADP ribosylation factor like GTPase 1 |
| P59998 | *ARPC4* | actin related protein 2/3 complex subunit 4 |
| Q676U5 | *ATG16L1* | autophagy related 16 like 1 |
| Q01814 | *ATP2B2* | ATPase plasma membrane Ca2+ transporting 2 |
| Q9UL15 | *BAG5* | BAG cochaperone 5 |
| P27708 | *CAD* | carbamoyl-phosphate synthetase 2, aspartate transcarbamylase, and dihydroorotase |
| Q9UQM7 | *CAMK2A* | calcium/calmodulin dependent protein kinase II alpha |
| Q13557 | *CAMK2D* | calcium/calmodulin dependent protein kinase II delta |
| Q14444 | *CAPRIN1* | cell cycle associated protein 1 |
| Q96JP9 | *CDHR1* | cadherin related family member 1 |
| Q00535 | *CDK5* | cyclin dependent kinase 5 |
| O75122 | *CLASP2* | cytoplasmic linker associated protein 2 |
| P09543 | *CNP* | 2',3'-cyclic nucleotide 3' phosphodiesterase |
| O14579 | *COPE* | COPI coat complex subunit epsilon |
| Q8N568 | *DCLK2* | doublecortin like kinase 2 |
| Q9NP97 | *DYNLRB1* | dynein light chain roadblock-type 1 |
| Q5JPH6 | *EARS2* | glutamyl-tRNA synthetase 2, mitochondrial |
| P68104 | *EEF1A1* | eukaryotic translation elongation factor 1 alpha 1 |
| Q8N336 | *ELMOD1* | ELMO domain containing 1 |
| Q15369 | *ELOC* | elongin C |
| P50402 | *EMD* | emerin |
| O75477 | *ERLIN1* | ER lipid raft associated 1 |
| O94905 | *ERLIN2* | ER lipid raft associated 2 |
| Q9BSJ8 | *ESYT1* | extended synaptotagmin 1 |
| Q9NZB2 | *FAM120A* | family with sequence similarity 120A |
| Q9NVF7 | *FBXO28* | F-box protein 28 |
| Q86UX7 | *FERMT3* | fermitin family member 3 |
| Q14318 | *FKBP8* | FKBP prolyl isomerase 8 |
| Q13045 | *FLII* | FLII actin remodeling protein |
| Q96I24 | *FUBP3* | far upstream element binding protein 3 |
| P51114 | *FXR1* | FMR1 autosomal homolog 1 |
| O94925 | *GLS* | glutaminase |
| P29992 | *GNA11* | G protein subunit alpha 11 |
| Q5JWF2 | *GNAS* | GNAS complex locus |
| P19086 | *GNAZ* | G protein subunit alpha z |
| O60741 | *HCN1* | hyperpolarization activated cyclic nucleotide gated potassium channel 1 |
| P52789 | *HK2* | hexokinase 2 |
| Q2TB90 | *HKDC1* | hexokinase domain containing 1 |
| P01892 | *HLA-A* | HLA class I histocompatibility antigen, A alpha chain |
| P31943 | *HNRNPH1* | heterogeneous nuclear ribonucleoprotein H1 |
| Q9BUJ2 | *HNRNPUL1* | heterogeneous nuclear ribonucleoprotein U like 1 |
| Q53GQ0 | *HSD17B12* | hydroxysteroid 17-beta dehydrogenase 12 |
| O43301 | *HSPA12A* | heat shock protein family A (Hsp70) member 12A |
| Q9Y4L1 | *HYOU1* | hypoxia up-regulated 1 |
| A1L0T0 | *ILVBL* | ilvB acetolactate synthase like |
| P20839 | *IMPDH1* | inosine monophosphate dehydrogenase 1 |
| Q6DN90 | *IQSEC1* | IQ motif and Sec7 domain ArfGEF 1 |
| Q06033 | *ITIH3* | inter-alpha-trypsin inhibitor heavy chain 3 |
| Q9Y287 | *ITM2B* | integral membrane protein 2B |
| Q9NQX7 | *ITM2C* | integral membrane protein 2C |
| Q14721 | *KCNB1* | potassium voltage-gated channel subfamily B member 1 |
| Q7Z4S6 | *KIF21A* | kinesin family member 21A |
| P32004 | *L1CAM* | L1 cell adhesion molecule |
| Q9BXB1 | *LGR4* | leucine rich repeat containing G protein-coupled receptor 4 |
| P49257 | *LMAN1* | lectin, mannose binding 1 |
| Q07954 | *LRP1* | LDL receptor related protein 1 |
| O95372 | *LYPLA2* | lysophospholipase 2 |
| P46821 | *MAP1B* | microtubule associated protein 1B |
| Q96JE9 | *MAP6* | microtubule associated protein 6 |
| C9JLW8 | *MCRIP1* | MAPK regulated corepressor interacting protein 1 |
| Q15773 | *MLF2* | myeloid leukemia factor 2 |
| Q13015 | *MLLT11* | MLLT11 transcription factor 7 cofactor |
| Q14168 | *MPP2* | membrane palmitoylated protein 2 |
| P11586 | *MTHFD1* | methylenetetrahydrofolate dehydrogenase, cyclohydrolase and formyltetrahydrofolate synthetase 1 |
| P35580 | *MYH10* | myosin heavy chain 10 |
| O14950 | *MYL12A* | myosin light chain 12A |
| P12829 | *MYL4* | myosin light chain 4 |
| P60660 | *MYL6* | myosin light chain 6 |
| Q9Y4I1 | *MYO5A* | myosin VA |
| Q9ULV0 | *MYO5B* | myosin VB |
| Q16718 | *NDUFA5* | NADH:ubiquinone oxidoreductase subunit A5 |
| O95168 | *NDUFB4* | NADH:ubiquinone oxidoreductase subunit B4 |
| O75306 | *NDUFS2* | NADH:ubiquinone oxidoreductase core subunit S2 |
| P49821 | *NDUFV1* | NADH:ubiquinone oxidoreductase core subunit V1 |
| P12036 | *NEFH* | neurofilament heavy |
| P46459 | *NSF* | N-ethylmaleimide sensitive factor, vesicle fusing ATPase |
| Q9BRJ7 | *NUDT16L1* | nudix hydrolase 16 like 1 |
| Q9BZF1 | *OSBPL8* | oxysterol binding protein like 8 |
| P11940 | *PABPC1* | poly(A) binding protein cytoplasmic 1 |
| Q13310 | *PABPC4* | poly(A) binding protein cytoplasmic 4 |
| Q6VY07 | *PACS1* | phosphofurin acidic cluster sorting protein 1 |
| Q96AQ6 | *PBXIP1* | PBX homeobox interacting protein 1 |
| O95206 | *PCDH8* | protocadherin 8 |
| P17858 | *PFKL* | phosphofructokinase, liver type |
| Q01813 | *PFKP* | phosphofructokinase, platelet |
| Q15149 | *PLEC* | plectin |
| P62191 | *PSMC1* | proteasome 26S subunit, ATPase 1 |
| P62333 | *PSMC6* | proteasome 26S subunit, ATPase 6 |
| P10586 | *PTPRF* | protein tyrosine phosphatase receptor type F |
| P47897 | *QARS1* | glutaminyl-tRNA synthetase 1 |
| Q15293 | *RCN1* | reticulocalbin 1 |
| Q14257 | *RCN2* | reticulocalbin 2 |
| Q96NR8 | *RDH12* | retinol dehydrogenase 12 |
| P62906 | *RPL10A* | ribosomal protein L10a |
| P30050 | *RPL12* | ribosomal protein L12 |
| P40429 | *RPL13A* | ribosomal protein L13a |
| P84098 | *RPL19* | ribosomal protein L19 |
| P46776 | *RPL27A* | ribosomal protein L27a |
| P62888 | *RPL30* | ribosomal protein L30 |
| P36578 | *RPL4* | ribosomal protein L4 |
| P62424 | *RPL7A* | ribosomal protein L7a |
| P62277 | *RPS13* | ribosomal protein S13 |
| P39019 | *RPS19* | ribosomal protein S19 |
| P15880 | *RPS2* | ribosomal protein S2 |
| Q8TD47 | *RPS4Y2* | ribosomal protein S4 Y-linked 2 |
| P62241 | *RPS8* | ribosomal protein S8 |
| O00442 | *RTCA* | RNA 3'-terminal phosphate cyclase |
| Q9Y3I0 | *RTCB* | RNA 2',3'-cyclic phosphate and 5'-OH ligase |
| P55735 | *SEC13* | SEC13 homolog, nuclear pore and COPII coat complex component |
| O15027 | *SEC16A* | SEC16 homolog A, endoplasmic reticulum export factor |
| Q15393 | *SF3B3* | splicing factor 3b subunit 3 |
| Q9NR46 | *SH3GLB2* | SH3 domain containing GRB2 like, endophilin B2 |
| Q02978 | *SLC25A11* | solute carrier family 25 member 11 |
| Q00325 | *SLC25A3* | solute carrier family 25 member 3 |
| P12236 | *SLC25A6* | solute carrier family 25 member 6 |
| Q9Y2P4 | *SLC27A6* | solute carrier family 27 member 6 |
| P17987 | *TCP1* | t-complex 1 |
| Q9NZ01 | *TECR* | trans-2,3-enoyl-CoA reductase |
| Q9UL33 | *TRAPPC2L* | trafficking protein particle complex 2 like |
| Q71U36 | *TUBA1A* | tubulin alpha 1a |
| P68366 | *TUBA4A* | tubulin alpha 4a |
| A6NHL2 | *TUBAL3* | tubulin alpha like 3 |
| P07437 | *TUBB* | tubulin beta class I |
| Q13509 | *TUBB3* | tubulin beta 3 class III |
| P04350 | *TUBB4A* | tubulin beta 4A class IVa |
| P68371 | *TUBB4B* | tubulin beta 4B class IVb |
| P23258 | *TUBG1* | tubulin gamma 1 |
| P49411 | *TUFM* | Tu translation elongation factor, mitochondrial |
| O95292 | *VAPB* | VAMP associated protein B and C |
| O94967 | *WDR47* | WD repeat domain 47 |
| P27348 | *YWHAQ* | 14-3-3 protein theta |


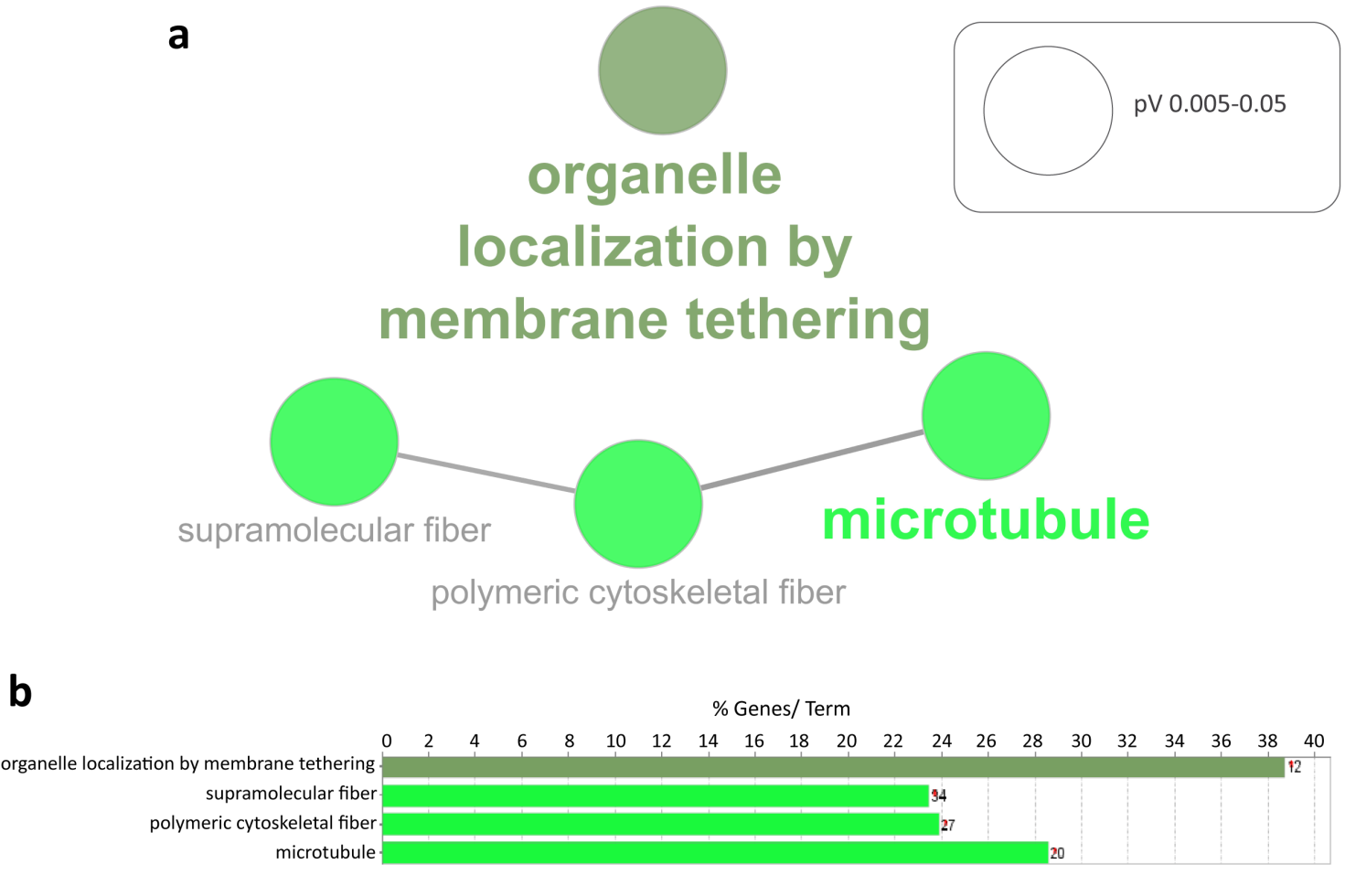


Supplementary Figure S3. ITM2B retinal interactome of the 140 common proteins purified with the mouse and the rabbit antibody. Ontology term enrichment and visualization were obtained using the ClueGo plug-in (Version 2.5.6) within the Cytoscape software (version 3.7.1) (a) GO-term network analysis (b) Functional clusters and pathways identified with enriched proteins. %Genes/Term corresponds to the proportion of genes enriched in the functional clusters. Bars with the same color belong to the same functional cluster.

**Supplementary Table S6.** GO-Terms enrichment analysis of the ITM2B retinal interactome of the 140 common proteins purified with both mouse and rabbit anti-ITM2B antibodies.

| **GO-Term** | **% Associated Genes** | **Nr Genes** | **Associated Proteins** |
| --- | --- | --- | --- |
| organelle localization by membrane tethering | 38,71 | 12,00 | ALB, ESYT1, KCNB1, NSF, TUBA1A, TUBA4A, TUBB, TUBB3, TUBB4A, TUBB4B, TUBG1, VAPB |
| supramolecular fiber | 23,45 | 34,00 | ACTB, ACTC1, ALB, ATP2B2, CAD, CDK5, CLASP2, DCLK2, DYNLRB1, EMD, FXR1, ITIH3, KIF21A, LMAN1, MAP1B, MAP6, MYH10, MYL12B, MYL4, MYO5A, NEFH, PBXIP1, PLEC, TCP1, TUBA1A, TUBA4A, TUBAL3, TUBB, TUBB3, TUBB4A, TUBB4B, TUBG1, WDR47, YWHAQ |
| polymeric cytoskeletal fiber | 23,89 | 27,00 | ACTB, ACTC1, ALB, CDK5, CLASP2, DCLK2, DYNLRB1, EMD, ITIH3, KIF21A, MAP1B, MAP6, MYO5A, NEFH, PBXIP1, PLEC, TCP1, TUBA1A, TUBA4A, TUBAL3, TUBB, TUBB3, TUBB4A, TUBB4B, TUBG1, WDR47, YWHAQ |
| Microtubule | 28,57 | 20,00 | CDK5, CLASP2, DCLK2, DYNLRB1, EMD, KIF21A, MAP1B, MAP6, MYO5A, PBXIP1, TCP1, TUBA1A, TUBA4A, TUBAL3, TUBB, TUBB3, TUBB4A, TUBB4B, TUBG1, WDR47 |

**
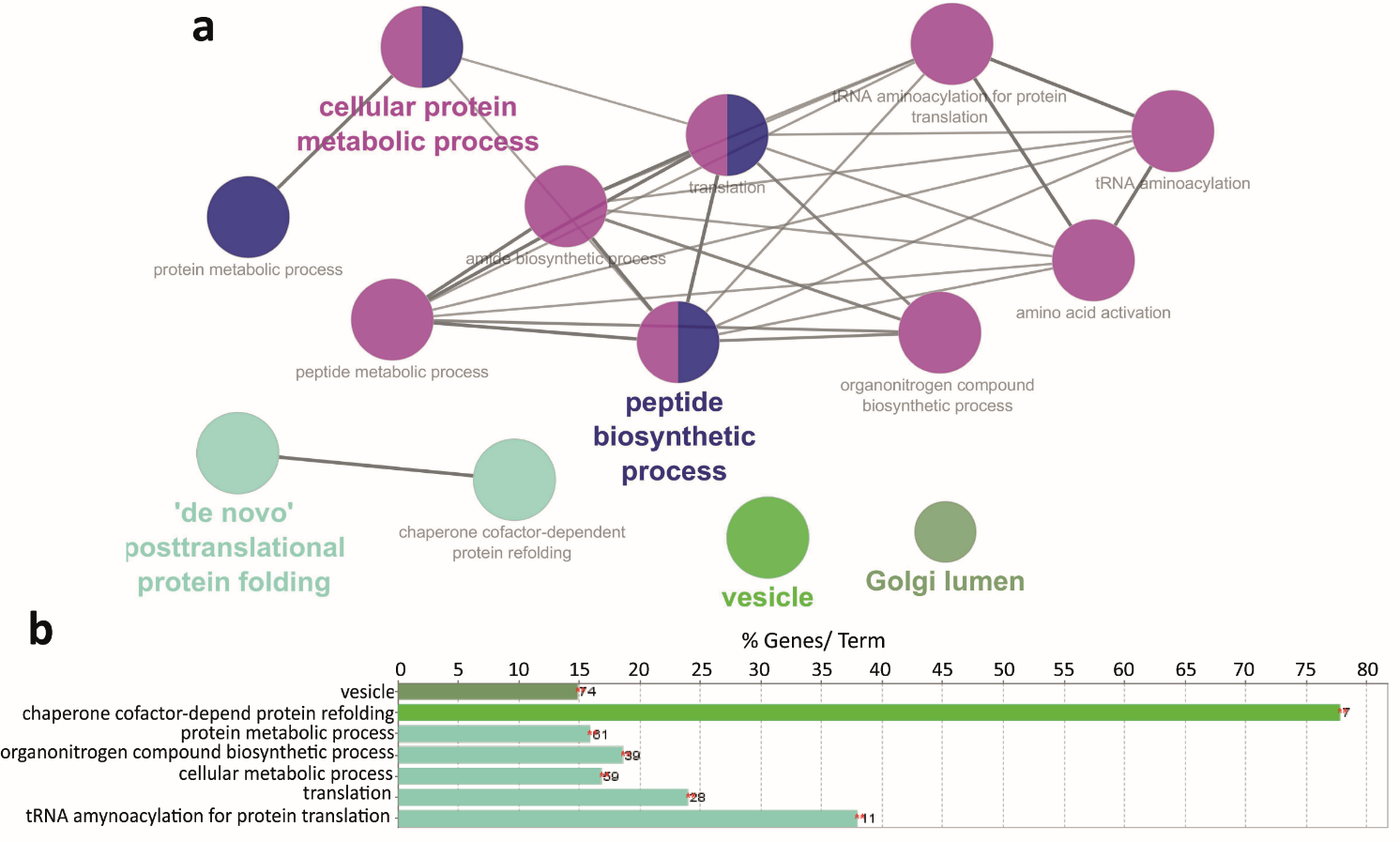
**

Supplementary Figure S4. ITM2B retinal interactome of the 114 proteins purified exclusively with the mouse antibody. Ontology term enrichment and visualization were obtained using the ClueGo plug-in (Version 2.5.6) within the Cytoscape software (version 3.7.1) (A) GO-term network analysis (B) Functional clusters and pathways enriched by upregulated proteins. %Genes/Term corresponds to the proportion of genes enriched in the functional clusters. Bars with the same color belong to the same functional cluster.


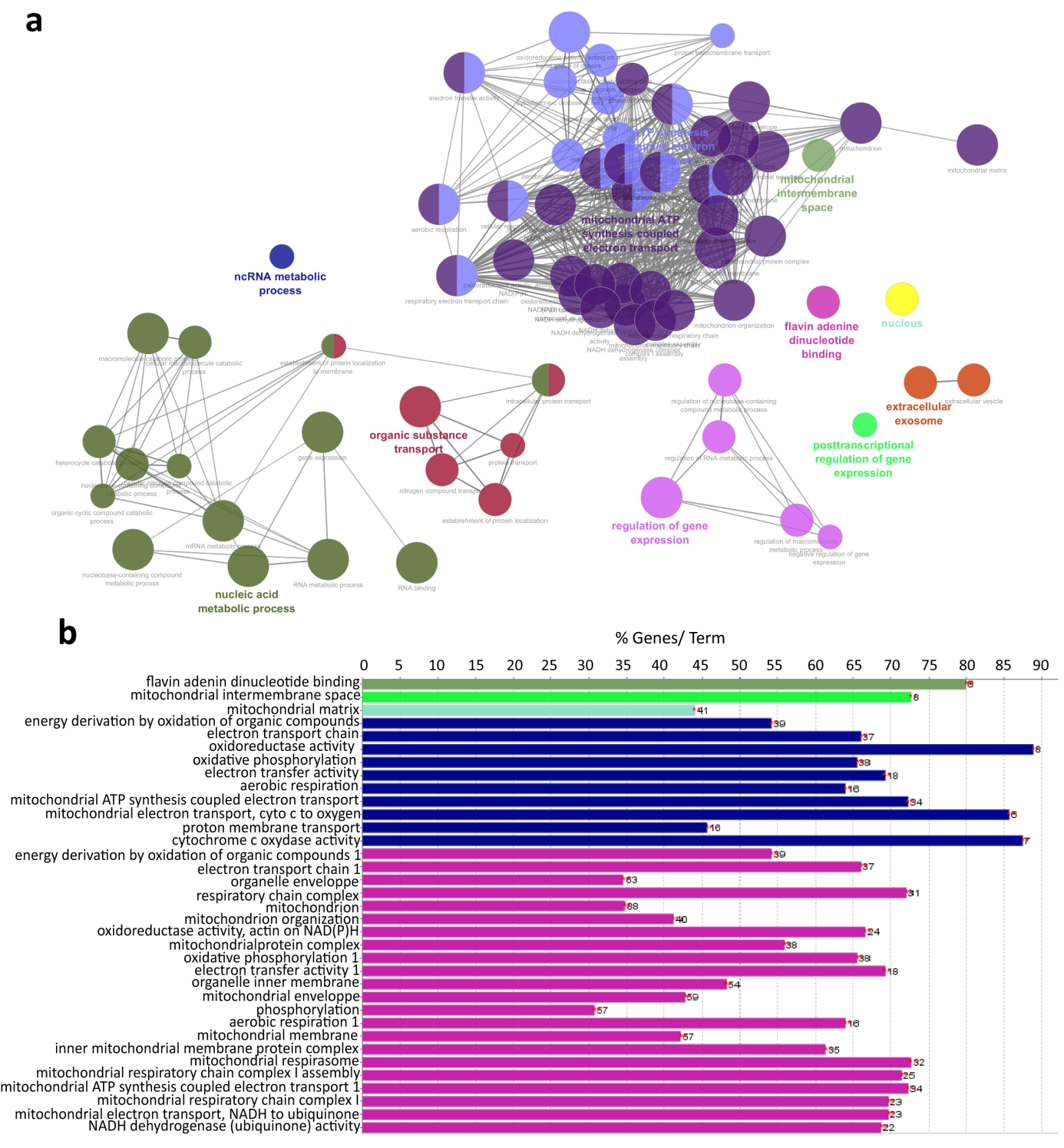


**Supplementary Figure S5.** ITM2B retinal interactome of the 220 proteins purified exclusively with the rabbit antibody. Ontology term enrichment and visualization were obtained using the ClueGo plug-in (Version 2.5.6) within the Cytoscape software (version 3.7.1). (a) GO-term network analysis (b) Functional clusters and pathways identified with enriched proteins. %Genes/Term corresponds to the proportion of genes enriched in the functional clusters. Bars with the same color belong to the same functional cluster.

**Supplementary Table S7.** GO-Terms enrichment analysis of the ITM2B retinal interactome purified with the mouse anti-ITM2B antibody (254 proteins (FC>2 and p-value<0.01)).

| **GO-Term** | **% Associated Genes** | **Nr Genes** | **Associated Proteins** |
| --- | --- | --- | --- |
| Microtubule | 42,86 | 30,00 | C11orf58, CCT3, CCT5, CCT8, CDK5, CKAP5, CLASP2, DCLK2, DYNC1H1, DYNLRB1, EMD, KIF21A, MAP1B, MAP6, MAPRE2, MYO5A, PBXIP1, RCC2, SRPRB, TCP1, TUBA1A, TUBA4A, TUBAL3, TUBB, TUBB2A, TUBB3, TUBB4A, TUBB4B, TUBG1, WDR47 |
| organonitrogen compound biosynthetic process | 33,33 | 70,00 | ABCE1, ACSL3, AIMP2, ATG16L1, ATP5PO, B4GAT1, CAD, DARS1, DCAKD, EARS2, EEF1A1, EEF1D, EEF1E1, EEF1G, EEF2, EIF3L, EIF4A1, GARS1, GLS, HSD17B12, HSPD1, IMPDH1, KARS1, L1CAM, LARS1, LMAN1, MARS1, MOGS, MTHFD1, MYO5A, NAMPT, PABPC1, PABPC4, PNPLA6, PRELP, PRPS1, QARS1, RACK1, RARS1, RPL10A, RPL12, RPL13A, RPL19, RPL23, RPL26, RPL27, RPL27A, RPL30, RPL4, RPL7A, RPS13, RPS16, RPS18, RPS19, RPS2, RPS20, RPS27A, RPS3, RPS4X, RPS4Y2, RPS8, RPSA, SLC25A12, SRM, TARS1, TARS3, TECR, TUFM, VAPB, VARS1 |
| peptide metabolic process | 37,40 | 49,00 | ABCE1, AIMP2, DARS1, EARS2, EEF1A1, EEF1D, EEF1E1, EEF1G, EEF2, EIF3L, EIF4A1, GARS1, HSPD1, KARS1, LARS1, MARS1, PABPC1, PABPC4, PCSK2, QARS1, RACK1, RARS1, RPL10A, RPL12, RPL13A, RPL19, RPL23, RPL26, RPL27, RPL27A, RPL30, RPL4, RPL7A, RPS13, RPS16, RPS18, RPS19, RPS2, RPS20, RPS27A, RPS3, RPS4X, RPS4Y2, RPS8, RPSA, TARS1, TARS3, TUFM, VARS1 |
| Translation | 41,03 | 48,00 | ABCE1, AIMP2, DARS1, EARS2, EEF1A1, EEF1D, EEF1E1, EEF1G, EEF2, EIF3L, EIF4A1, GARS1, HSPD1, KARS1, LARS1, MARS1, PABPC1, PABPC4, QARS1, RACK1, RARS1, RPL10A, RPL12, RPL13A, RPL19, RPL23, RPL26, RPL27, RPL27A, RPL30, RPL4, RPL7A, RPS13, RPS16, RPS18, RPS19, RPS2, RPS20, RPS27A, RPS3, RPS4X, RPS4Y2, RPS8, RPSA, TARS1, TARS3, TUFM, VARS1 |

Supplementary Table S8. List representing 124 proteins which are in common between the 440 proteins identified by LC-MS/MS and purified with the mouse anti-ITM2B antibody (FC>2 and p-value<0.05) and the 511 rat cerebral proteins identified in a previous study[^1^](#_ENREF_1).

| **Uniprot accession** | **Gene name** | **Protein name** |
| --- | --- | --- |

| P06238 | *A2m* | Alpha-2-macroglobulin |
| --- | --- | --- |
| P60711 | *Actb* | Actin, cytoplasmic 1 |
| P68035 | *Actc1* | Actin, alpha cardiac muscle 1 |
| O70511 | *Ank3* | Ankyrin-3 |
| P84092 | *Ap2m1* | AP-2 complex subunit mu |
| P06687 | *Atp1a3* | Sodium/potassium-transporting ATPase subunit alpha-3 |
| P11507 | *Atp2a2* | Sarcoplasmic/endoplasmic reticulum calcium ATPase 2 |
| P11506 | *Atp2b2* | Calcium-transporting ATPase Plasma membrane calcium-transporting ATPase 2 |
| Q64542 | *Atp2b4* | Calcium-transporting ATPase Plasma membrane calcium-transporting ATPase 4 |
| P35435 | *Atp5f1c* | ATP synthase subunit gamma, mitochondrial |
| P35434 | *Atp5f1d* | ATP synthase subunit delta, mitochondrial |
| Q9JJW3 | *Atp5md* | ATP synthase membrane subunit DAPIT, mitochondrial |
| P31399 | *Atp5pd* | ATP synthase subunit d, mitochondrial |
| Q06647 | *Atp5po* | ATP synthase subunit O, mitochondrial |
| P01026 | *C3* | Complement C3 |
| Q66HR5 | *Calcoco1* | Calcium-binding and coiled-coil domain-containing protein 1 |
| P11275 | *Camk2a* | Calcium/calmodulin-dependent protein kinase type II subunit alpha |
| P15791 | *Camk2d* | Calcium/calmodulin-dependent protein kinase type II |
| Q3T1K5 | *Capza2* | F-actin-capping protein subunit alpha-2 |
| Q5XI32 | *Capzb* | F-actin-capping protein subunit beta |
| Q6P502 | *Cct3* | T-complex protein 1 subunit gamma |
| Q7TPB1 | *Cct4* | T-complex protein 1 subunit delta |
| Q68FQ0 | *Cct5* | T-complex protein 1 subunit epsilon |
| Q03114 | *Cdk5* | Cyclin-dependent-like kinase 5 |
| Q99JD4 | *Clasp2* | CLIP-associating protein 2 |
| P08081 | *Clta* | Clathrin light chain A |
| P13233 | *Cnp* | 2',3'-cyclic-nucleotide 3'-phosphodiesterase |
| P12075 | *Cox5b* | Cytochrome c oxidase subunit 5B, mitochondrial |
| P35171 | *Cox7a2* | Cytochrome c oxidase subunit 7A2, mitochondrial |
| O08875 | *Dclk1* | Serine/threonine-protein kinase DCLK1 |
| P38650 | *Dync1h1* | Cytoplasmic dynein 1 heavy chain 1 Dynein |
| P62630 | *Eef1a1* | Elongation factor 1-alpha 1 |
| Q68FR6 | *Eef1g* | Elongation factor 1-gamma |
| P05197 | *Eef2* | Elongation factor 2 |
| B5DEH2 | *Erlin2* | Erlin-2 |
| Q5XI81 | *Fxr1* | Fragile X mental retardation syndrome-related protein 1 |
| P97874 | *Gak* | Cyclin-G-associated kinase |
| P04897 | *Gnai2* | Guanine nucleotide-binding protein G(i) subunit alpha-2 |
| P63095 | *Gnas* | Guanine nucleotide-binding protein G(s) subunit alpha isoforms short |
| P19627 | *Gnaz* | Guanine nucleotide-binding protein G(z) subunit alpha |
| Q64428 | *Hadha* | Trifunctional enzyme subunit alpha, mitochondrial |
| Q8VHV7 | *Hnrnph1* | Heterogeneous nuclear ribonucleoprotein H RRM domain-containing protein |
| P82995 | *Hsp90aa1* | Heat shock protein HSP 90-alpha |
| P34058 | *Hsp90ab1* | Heat shock protein HSP 90-beta |
| Q66HD0 | *Hsp90b1* | Endoplasmin |
| O88600 | *Hspa4* | Heat shock 70 kDa protein 4L |
| P06761 | *Hspa5* | Endoplasmic reticulum chaperone BiP |
| P63039 | *Hspa8* | Heat shock cognate 71 kDa protein |
| P63039 | *Hspd1* | 60 kDa heat shock protein, mitochondrial |
| Q63617 | *Hyou1* | Hypoxia up-regulated protein 1 |
| Q3KR86 | *Immt* | MICOS complex subunit Mic60 |
| P23565 | *Ina* | Alpha-internexin |
| Q63416 | *Itih3* | Inter-alpha-trypsin inhibitor heavy chain H3 |
| Q2PQA9 | *Kif5b* | Kinesin-1 heavy chain |
| O08873 | *Madd* | MAP kinase-activating death domain protein |
| P15205 | *Map1b* | Microtubule-associated protein 1B |
| Q63560 | *Map6* | Microtubule-associated protein 6 |
| Q9JLT0 | *Myh10* | Myosin-10 |
| Q64119 | *Myl6* | Myosin light polypeptide 6 |
| Q9QYF3 | *Myo5a* | Unconventional myosin-Va |
| P85969 | *Napb* | Beta-soluble NSF attachment protein |
| P13596 | *Ncam1* | Neural cell adhesion molecule 1 |
| Q561S0 | *Ndufa10* | NADH dehydrogenase [ubiquinone] 1 alpha subcomplex subunit 10, mitochondrial |
| Q641Y2 | *Ndufs2* | NADH dehydrogenase [ubiquinone] iron-sulfur protein 2, mitochondrial |
| P19234 | *Ndufv2* | NADH dehydrogenase [ubiquinone] flavoprotein 2, mitochondrial |
| P16884 | *Nefh* | Neurofilament heavy polypeptide |
| P19527 | *Nefl* | Neurofilament light polypeptide |
| P12839 | *Nefm* | Neurofilament medium polypeptide |
| Q9QUL6 | *Nsf* | Vesicle-fusing ATPase |
| P04785 | *P4hb* | Protein disulfide-isomerase |
| Q6AYD3 | *Pa2g4* | Proliferation-associated protein 2G4 |
| Q9EPH8 | *Pabpc1* | Polyadenylate-binding protein 1 |
| P47858 | *Pfkm* | ATP-dependent 6-phosphofructokinase, muscle type 6-phosphofructokinase |
| P47860 | *Pfkp* | ATP-dependent 6-phosphofructokinase, platelet type |
| P62963 | *Pfn1* | Profilin-1 Profilin |
| O08662 | *Pi4ka* | Phosphatidylinositol 4-kinase alpha |
| P30427 | *Plec* | Plectin |
| P10111 | *Ppia* | Peptidyl-prolyl cis-trans isomerase A |
| Q4FZT9 | *Psmd2* | 26S proteasome non-ATPase regulatory subunit 2 |
| P53534 | *Pygb* | Alpha-1,4 glucan phosphorylase Glycogen phosphorylase, brain form |
| P61107 | *Rab14* | Ras-related protein Rab-14 |
| P63245 | *Rack1* | Receptor of activated protein C kinase 1 |
| Q62703 | *Rcn2* | Reticulocalbin-2 |
| P62914 | *Rpl11* | 60S ribosomal protein L11 |
| P23358 | *Rpl12* | 60S ribosomal protein L12 |
| P47198 | *Rpl22* | 60S ribosomal protein L22 |
| P61354 | *Rpl27* | 60S ribosomal protein L27 |
| P50878 | *Rpl4* | 60S ribosomal protein L4 |
| P21533 | *Rpl6* | 60S ribosomal protein L6 |
| P19945 | *Rplp0* | 60S acidic ribosomal protein P0 |
| P62250 | *Rps16* | 40S ribosomal protein S16 |
| P60868 | *Rps20* | 40S ribosomal protein S20 |
| P62909 | *Rps3* | 40S ribosomal protein S3 |
| P49242 | *Rps3a* | 40S ribosomal protein S3a |
| P62703 | *Rps4x* | 40S ribosomal protein S4, X isoform |
| Q6AYT3 | *Rtcb* | RNA-splicing ligase RtcB homolog |
| Q6P799 | *Sars1* | Serine--tRNA ligase, cytoplasmic |
| Q5PPJ9 | *Sh3glb2* | Endophilin-B2 |
| Q63633 | *Slc12a5* | Solute carrier family 12 member 5 |
| P97700 | *Slc25a11* | Mitochondrial 2-oxoglutarate/malate carrier protein |
| P16036 | *Slc25a3* | Phosphate carrier protein, mitochondrial |
| Q05962 | *Slc25a4* | ADP/ATP translocase 1 |
| Q09073 | *Slc25a5* | ADP/ATP translocase 2 |
| P11167 | *Slc2a1* | Solute carrier family 2, facilitated glucose transporter member 1 |
| Q9QXY2 | *Srcin1* | SRC kinase signaling inhibitor 1 |
| P61765 | *Stxbp1* | Syntaxin-binding protein 1 |
| Q7TP47 | *Syncrip* | Heterogeneous nuclear ribonucleoprotein Q |
| P12346 | *Tf* | Serotransferrin |
| P70566 | *Tmod2* | Tropomodulin-2 |
| P68370 | *Tuba1a* | Tubulin alpha-1A chain |
| Q5XIF6 | *Tuba4a* | Tubulin alpha-4A chain |
| Q6AY56 | *Tuba8* | Tubulin alpha-8 chain |
| P85108 | *Tubb2a* | Tubulin beta-2A chain |
| Q3KRE8 | *Tubb2b* | Tubulin beta-2B chain |
| Q4QRB4 | *Tubb3* | Tubulin beta-3 chain |
| Q6P9T8 | *Tubb4b* | Tubulin beta-4B chain Tubulin beta chain |
| P69897 | *Tubb5* | Tubulin beta chain Tubulin beta-5 chain |
| P85834 | *Tufm* | Elongation factor Tu Elongation factor Tu, mitochondrial |
| Q7TQ16 | *Uqcrq* | Cytochrome b-c1 complex subunit 8 |
| Q04462 | *Vars1* | Valine--tRNA ligase |
| Q793F9 | *Vps4a* | Vacuolar protein sorting-associated protein 4A |
| Q9ERH3 | *Wdr7* | WD repeat-containing protein 7 |
| P68255 | *Ywhaq* | 14-3-3 protein theta |
| P63102 | *Ywhaz* | 14-3-3 protein zeta/delta |

Supplementary Table S9. GO-Terms enrichment analysis of the ITM2B retinal interactome purified with the rabbit anti-ITM2B (360 proteins (FC>2 and p-value<0.01)).

| **GO-Term** | **% Associated Genes** | **Nr Genes** | **Associated Proteins** |
| --- | --- | --- | --- |
| Mitochondrion | 43,08 | 109,00 | AARS2, ABCE1, ABHD11, ACAD9, ACADM, ACADSB, ACSF3, AK4, ALDH18A1, ANK2, ARL2, ATAD3A, ATP5F1A, ATP5F1C, ATP5MD, ATP5PF, BAG5, BSG, CAMK2A, CNP, COQ8B, COX2, COX4I1, COX5A, COX5B, COX6C, COX7A2, COX7A2L, CRYAB, CYC1, CYP27A1, DDX6, DGKE, DNAJA3, DNM1L, EARS2, EEF1A2, FKBP8, FOXRED1, GFM2, GIT1, GLS, GNAS, GUF1, HADHA, HADHB, HIGD1A, HK2, HKDC1, IARS2, IDH2, IDH3A, IMMT, KMT2D, LDHD, LIG3, MCCC1, MCCC2, MFF, MTCH2, MTHFD1, MTHFD1L, NDUFA10, NDUFA13, NDUFA2, NDUFA3, NDUFA5, NDUFA6, NDUFA7, NDUFA9, NDUFB10, NDUFB4, NDUFB5, NDUFB6, NDUFB7, NDUFB8, NDUFB9, NDUFS1, NDUFS2, NDUFS3, NDUFS4, NDUFS5, NDUFS6, NDUFS7, NDUFS8, NDUFV1, NDUFV2, NDUFV3, NUBPL, OAT, PC, PPOX, PYCR2, QARS1, SDHA, SLC25A11, SLC25A3, SLC25A6, STOML2, STXBP1, SUCLA2, SUCLG1, TUFM, UQCRC1, UQCRC2, UQCRQ, VDAC2, YARS2, YWHAQ |
| myosin complex | 80,00 | 12,00 | [MYH10, MYH14, MYH9, MYL12B, MYL4, MYL6, MYL9, MYO18A, MYO1C, MYO5A, MYO5B, MYO7A |
| mitochondrial matrix | 50,54 | 47,00 | [ABCE1, ACADM, ACADSB, ACSF3, AK4, ARL2, ATAD3A, ATP5F1A, ATP5F1C, ATP5PF, CYP27A1, DGKE, DNAJA3, DNM1L, EARS2, GFM2, GLS, GUF1, HADHA, HADHB, IARS2, IDH2, IDH3A, LDHD, MCCC1, MCCC2, MTHFD1L, NDUFA10, NDUFA7, NDUFA9, NDUFB8, NDUFS1, NDUFS2, NDUFS3, NDUFS7, NDUFS8, NUBPL, OAT, PC, PYCR2, QARS1, SUCLA2, SUCLG1, TUFM, UQCRC2, VDAC2, YARS2 |
| mitochondrial protein complex | 63,24 | 43,00 | ATP5F1A, ATP5F1C, ATP5MD, ATP5PF, COX4I1, COX5A, CYC1, FOXRED1, HADHA, IMMT, MCCC1, MCCC2, NDUFA10, NDUFA13, NDUFA2, NDUFA3, NDUFA5, NDUFA6, NDUFA7, NDUFA9, NDUFB10, NDUFB4, NDUFB5, NDUFB6, NDUFB7, NDUFB8, NDUFB9, NDUFS1, NDUFS2, NDUFS3, NDUFS4, NDUFS5, NDUFS6, NDUFS7, NDUFS8, NDUFV1, NDUFV2, NDUFV3, SDHA, SLC25A6, UQCRC1, UQCRC2, UQCRQ |
| oxidative phosphorylation | 72,41 | 42,00 | ATP5F1A, ATP5F1C, ATP5PF, COX2, COX4I1, COX5A, COX5B, COX6C, COX7A2L, CYC1, LDHD, NDUFA10, NDUFA13, NDUFA2, NDUFA3, NDUFA5, NDUFA6, NDUFA7, NDUFA9, NDUFB10, NDUFB4, NDUFB5, NDUFB6, NDUFB7, NDUFB8, NDUFB9, NDUFS1, NDUFS2, NDUFS3, NDUFS4, NDUFS5, NDUFS6, NDUFS7, NDUFS8, NDUFV1, NDUFV2, NDUFV3, SDHA, STOML2, UQCRC1, UQCRC2, UQCRQ |
| electron transfer activity | 73,08 | 19,00 | COX2, COX4I1, COX5A, COX5B, COX6C, COX7A2, COX7A2L, CYC1, KMT2D, LDHD, NDUFS1, NDUFS2, NDUFS3, NDUFS6, NDUFV2, SDHA, UQCRC1, UQCRQ, VDAC2 |
| organelle inner membrane | 56,25 | 63,00 | ACAD9, ALDH18A1, ATAD3A, ATP5F1A, ATP5F1C, ATP5MD, ATP5PF, CNP, COQ8B, COX2, COX4I1, COX5A, COX5B, COX6C, COX7A2, COX7A2L, CYC1, CYP27A1, EMD, FOXRED1, GUF1, HADHA, HADHB, HIGD1A, IMMT, LDHD, MTCH2, NDUFA10, NDUFA13, NDUFA2, NDUFA3, NDUFA5, NDUFA6, NDUFA7, NDUFA9, NDUFB10, NDUFB4, NDUFB5, NDUFB6, NDUFB7, NDUFB8, NDUFB9, NDUFS1, NDUFS2, NDUFS3, NDUFS4, NDUFS5, NDUFS6, NDUFS7, NDUFS8, NDUFV1, NDUFV2, NDUFV3, PPOX, SDHA, SLC25A11, SLC25A3, SLC25A6, STOML2, TMPO, UQCRC1, UQCRC2, UQCRQ |
| cellular respiration | 67,74 | 42,00 | COX2, COX4I1, COX5A, COX5B, COX6C, COX7A2L, CYC1, IDH2, IDH3A, LDHD, NDUFA10, NDUFA13, NDUFA2, NDUFA3, NDUFA5, NDUFA6, NDUFA7, NDUFA9, NDUFB10, NDUFB4, NDUFB5, NDUFB6, NDUFB7, NDUFB8, NDUFB9, NDUFS1, NDUFS2, NDUFS3, NDUFS4, NDUFS5, NDUFS6, NDUFS7, NDUFS8, NDUFV1, NDUFV2, NDUFV3, SDHA, SUCLA2, SUCLG1, UQCRC1, UQCRC2, UQCRQ |
| mitochondrial envelope | 51,45 | 71,00 | ACAD9, ACADM, ALDH18A1, ARL2, ATAD3A, ATP5F1A, ATP5F1C, ATP5MD, ATP5PF, CNP, COQ8B, COX2, COX4I1, COX5A, COX5B, COX6C, COX7A2, COX7A2L, CYC1, CYP27A1, DNM1L, FKBP8, FOXRED1, GNAS, GUF1, HADHA, HADHB, HIGD1A, HK2, HKDC1, IMMT, KMT2D, LDHD, MFF, MTCH2, NDUFA10, NDUFA13, NDUFA2, NDUFA3, NDUFA5, NDUFA6, NDUFA7, NDUFA9, NDUFB10, NDUFB4, NDUFB5, NDUFB6, NDUFB7, NDUFB8, NDUFB9, NDUFS1, NDUFS2, NDUFS3, NDUFS4, NDUFS5, NDUFS6, NDUFS7, NDUFS8, NDUFV1, NDUFV2, NDUFV3, PPOX, SDHA, SLC25A11, SLC25A3, SLC25A6, STOML2, UQCRC1, UQCRC2, UQCRQ, VDAC2 |
| mitochondrial membrane | 50,37 | 68,00 | ACAD9, ACADM, ALDH18A1, ATAD3A, ATP5F1A, ATP5F1C, ATP5MD, ATP5PF, CNP, COQ8B, COX2, COX4I1, COX5A, COX5B, COX6C, COX7A2, COX7A2L, CYC1, CYP27A1, DNM1L, FKBP8, FOXRED1, GUF1, HADHA, HADHB, HIGD1A, HK2, HKDC1, IMMT, LDHD, MFF, MTCH2, NDUFA10, NDUFA13, NDUFA2, NDUFA3, NDUFA5, NDUFA6, NDUFA7, NDUFA9, NDUFB10, NDUFB4, NDUFB5, NDUFB6, NDUFB7, NDUFB8, NDUFB9, NDUFS1, NDUFS2, NDUFS3, NDUFS4, NDUFS5, NDUFS6, NDUFS7, NDUFS8, NDUFV1, NDUFV2, NDUFV3, PPOX, SDHA, SLC25A11, SLC25A3, SLC25A6, STOML2, UQCRC1, UQCRC2, UQCRQ, VDAC2 |
| inner mitochondrial membrane protein complex | 70,18 | 40,00 | ATP5F1A, ATP5F1C, ATP5MD, ATP5PF, COX4I1, COX5A, CYC1, FOXRED1, IMMT, NDUFA10, NDUFA13, NDUFA2, NDUFA3, NDUFA5, NDUFA6, NDUFA7, NDUFA9, NDUFB10, NDUFB4, NDUFB5, NDUFB6, NDUFB7, NDUFB8, NDUFB9, NDUFS1, NDUFS2, NDUFS3, NDUFS4, NDUFS5, NDUFS6, NDUFS7, NDUFS8, NDUFV1, NDUFV2, NDUFV3, SDHA, SLC25A6, UQCRC1, UQCRC2, UQCRQ |
| mitochondrial respirasome | 81,82 | 36,00 | COX4I1, COX5A, COX7A2, COX7A2L, CYC1, FOXRED1, NDUFA10, NDUFA13, NDUFA2, NDUFA3, NDUFA5, NDUFA6, NDUFA7, NDUFA9, NDUFB10, NDUFB4, NDUFB5, NDUFB6, NDUFB7, NDUFB8, NDUFB9, NDUFS1, NDUFS2, NDUFS3, NDUFS4, NDUFS5, NDUFS6, NDUFS7, NDUFS8, NDUFV1, NDUFV2, NDUFV3, SDHA, UQCRC1, UQCRC2, UQCRQ |
| mitochondrial inner membrane | 59,80 | 61,00 | ACAD9, ALDH18A1, ATAD3A, ATP5F1A, ATP5F1C, ATP5MD, ATP5PF, CNP, COQ8B, COX2, COX4I1, COX5A, COX5B, COX6C, COX7A2, COX7A2L, CYC1, CYP27A1, FOXRED1, GUF1, HADHA, HADHB, HIGD1A, IMMT, LDHD, MTCH2, NDUFA10, NDUFA13, NDUFA2, NDUFA3, NDUFA5, NDUFA6, NDUFA7, NDUFA9, NDUFB10, NDUFB4, NDUFB5, NDUFB6, NDUFB7, NDUFB8, NDUFB9, NDUFS1, NDUFS2, NDUFS3, NDUFS4, NDUFS5, NDUFS6, NDUFS7, NDUFS8, NDUFV1, NDUFV2, NDUFV3, PPOX, SDHA, SLC25A11, SLC25A3, SLC25A6, STOML2, UQCRC1, UQCRC2, UQCRQ |
| mitochondrial respiratory chain complex I assembly | 82,86 | 29,00 | ACAD9, FOXRED1, NDUFA10, NDUFA13, NDUFA2, NDUFA3, NDUFA5, NDUFA6, NDUFA7, NDUFA9, NDUFB10, NDUFB4, NDUFB5, NDUFB6, NDUFB7, NDUFB8, NDUFB9, NDUFS1, NDUFS2, NDUFS3, NDUFS4, NDUFS5, NDUFS6, NDUFS7, NDUFS8, NDUFV1, NDUFV2, NDUFV3, NUBPL |
| mitochondrial ATP synthesis coupled electron transport | 80,85 | 38,00 | COX2, COX4I1, COX5A, COX5B, COX6C, COX7A2L, CYC1, LDHD, NDUFA10, NDUFA13, NDUFA2, NDUFA3, NDUFA5, NDUFA6, NDUFA7, NDUFA9, NDUFB10, NDUFB4, NDUFB5, NDUFB6, NDUFB7, NDUFB8, NDUFB9, NDUFS1, NDUFS2, NDUFS3, NDUFS4, NDUFS5, NDUFS6, NDUFS7, NDUFS8, NDUFV1, NDUFV2, NDUFV3, SDHA, UQCRC1, UQCRC2, UQCRQ |
| mitochondrial respiratory chain complex I | 81,82 | 27,00 | FOXRED1, NDUFA10, NDUFA13, NDUFA2, NDUFA3, NDUFA5, NDUFA6, NDUFA7, NDUFA9, NDUFB10, NDUFB4, NDUFB5, NDUFB6, NDUFB7, NDUFB8, NDUFB9, NDUFS1, NDUFS2, NDUFS3, NDUFS4, NDUFS5, NDUFS6, NDUFS7, NDUFS8, NDUFV1, NDUFV2, NDUFV3 |
| mitochondrial electron transport, NADH to ubiquinone | 81,82 | 27,00 | LDHD, NDUFA10, NDUFA13, NDUFA2, NDUFA3, NDUFA5, NDUFA6, NDUFA7, NDUFA9, NDUFB10, NDUFB4, NDUFB5, NDUFB6, NDUFB7, NDUFB8, NDUFB9, NDUFS1, NDUFS2, NDUFS3, NDUFS4, NDUFS5, NDUFS6, NDUFS7, NDUFS8, NDUFV1, NDUFV2, NDUFV3 |
| NADH dehydrogenase (ubiquinone) activity | 81,25 | 26,00 | NDUFA10, NDUFA13, NDUFA2, NDUFA3, NDUFA5, NDUFA6, NDUFA7, NDUFA9, NDUFB10, NDUFB4, NDUFB5, NDUFB6, NDUFB7, NDUFB8, NDUFB9, NDUFS1, NDUFS2, NDUFS3, NDUFS4, NDUFS5, NDUFS6, NDUFS7, NDUFS8, NDUFV1, NDUFV2, NDUFV3 |

Supplementary Table S10. List representing 150 proteins which are in common between the 615 proteins identified by LC-MS/MS and purified with the rabbit anti-ITM2B antibody (FC>2 and p-value<0.05) and the 511 rat cerebral proteins identified in a previous study[^1^](#_ENREF_1).

| **Uniprot accession** | **Gene name** | **Protein name** |
| --- | --- | --- |

| P06238 | *A2m* | Alpha-2-macroglobulin-P Alpha-2-macroglobulin |
| --- | --- | --- |
| Q6KC51 | *Ablim2* | Actin-binding LIM protein 2 |
| P60711 | *Actb* | Actin, cytoplasmic 1 |
| P68035 | *Actc1* | Actin, alpha cardiac muscle 1 |
| Q63028 | *Add1* | Alpha-adducin |
| O08838 | *Amph* | Amphiphysin BAR domain-containing protein |
| O70511 | *Ank3* | Ankyrin-3 |
| P06685 | *Atp1a1* | Sodium/potassium-transporting ATPase subunit alpha-1 |
| P06687 | *Atp1a3* | Sodium/potassium-transporting ATPase subunit alpha-3 |
| P07340 | *Atp1b1* | Sodium/potassium-transporting ATPase subunit beta-1 |
| P13638 | *Atp1b2* | Sodium/potassium-transporting ATPase subunit beta-2 |
| P11505 | *Atp2b1* | Plasma membrane calcium-transporting ATPase 1 |
| P11506 | *Atp2b2* | Plasma membrane calcium-transporting ATPase 2 |
| Q64542 | *Atp2b4* | Plasma membrane calcium-transporting ATPase 4 |
| P15999 | *Atp5f1a* | ATP synthase subunit alpha, mitochondrial ATP synthase subunit alpha |
| P10719 | *Atp5f1b* | ATP synthase subunit beta, mitochondrial ATP synthase subunit beta |
| P35435 | *Atp5f1c* | ATP synthase subunit gamma ATP synthase subunit gamma, mitochondrial |
| Q9JJW3 | *Atp5md* | ATP synthase membrane subunit DAPIT, mitochondrial |
| P31399 | *Atp5pd* | ATP synthase subunit d, mitochondrial |
| P21571 | *Atp5pf* | ATP synthase-coupling factor 6, mitochondrial |
| Q06647 | *Atp5po* | ATP synthase subunit O, mitochondrial |
| P25286 | *Atp6v0a1* | V-type proton ATPase 116 kDa subunit a isoform 1 |
| P62815 | *Atp6v1b2* | V-type proton ATPase subunit B, brain isoform |
| O08839 | *Bin1* | Myc box-dependent-interacting protein 1 |
| P01026 | *C3* | Complement C3 |
| P54290 | *Cacna2d1* | Voltage-dependent calcium channel subunit alpha-2/delta-1 |
| P62161 | *Calm1* | Calmodulin-1 |
| P11275 | *Camk2a* | Calcium/calmodulin-dependent protein kinase type II subunit alpha |
| P08413 | *Camk2b* | Calcium/calmodulin-dependent protein kinase type II subunit beta |
| P15791 | *Camk2d* | Calcium/calmodulin-dependent protein kinase type II subunit delta |
| P11730 | *Camk2g* | Calcium/calmodulin-dependent protein kinase type II subunit gamma |
| Q63092 | *Camkv* | CaM kinase-like vesicle-associated protein |
| P97536 | *Cand1* | Cullin-associated NEDD8-dissociated protein 1 |
| Q3T1K5 | *Capza2* | F-actin-capping protein subunit alpha-2 F-actin-capping protein subunit alpha |
| Q5XI32 | *Capzb* | F-actin-capping protein subunit beta |
| Q7TPB1 | *Cct4* | T-complex protein 1 subunit delta |
| Q68FQ0 | *Cct5* | T-complex protein 1 subunit epsilon |
| Q03114 | *Cdk5* | Cyclin-dependent-like kinase 5 |
| Q99JD4 | *Clasp2* | CLIP-associating protein 2 TOG domain-containing protein |
| P13233 | *Cnp* | 2',3'-cyclic-nucleotide 3'-phosphodiesterase |
| Q63198 | *Cntn1* | Contactin-1 |
| P97536 | *Cntnap1* | Contactin-associated protein 1 |
| P10888 | *Cox4i1* | Cytochrome c oxidase subunit 4 isoform 1, mitochondrial |
| P11240 | *Cox5a* | Cytochrome c oxidase subunit 5A, mitochondrial |
| P12075 | *Cox5b* | Cytochrome c oxidase subunit 5B, mitochondrial |
| P35171 | *Cox7a2* | Cytochrome c oxidase subunit 7A2, mitochondrial |
| P19139 | *Csnk2a1* | Casein kinase II subunit alpha |
| Q9WU82 | *Ctnnb1* | Catenin beta-1 |
| O08875 | *Dclk1* | Serine/threonine-protein kinase DCLK1 |
| Q01205 | *Dlst* | Dihydrolipoyllysine-residue succinyltransferase component of 2-oxoglutarate dehydrogenase complex, mitochondrial |
| P21575 | *Dnm1* | Dynamin-1 |
| O35303 | *Dnm1l* | Dynamin-1-like protein |
| P38650 | *Dync1h1* | Cytoplasmic dynein 1 heavy chain 1 |
| P62630 | *Eef1a1* | Elongation factor 1-alpha 1 |
| P05197 | *Eef2* | Elongation factor 2 |
| B5DEH2 | *Erlin2* | Erlin-2 |
| Q5XI81 | *Fxr1* | Fragile X mental retardation syndrome-related protein 1 |
| P97874 | *Gak* | Cyclin-G-associated kinase |
| P04897 | *Gnai2* | Guanine nucleotide-binding protein G(i) subunit alpha-2 |
| P59215 | *Gnao1* | Guanine nucleotide-binding protein G(o) subunit alpha |
| P63095 | *Gnas* | Guanine nucleotide-binding protein G(s) subunit alpha isoforms short |
| P19627 | *Gnaz* | Guanine nucleotide-binding protein G(z) subunit alpha |
| Q68FP1 | *Gsn* | Gelsolin |
| Q64428 | *Hadha* | Trifunctional enzyme subunit alpha, mitochondrial |
| Q60587 | *Hadhb* | Trifunctional enzyme subunit beta, mitochondrial |
| Q794E4 | *Hnrnpf* | Heterogeneous nuclear ribonucleoprotein F |
| Q8VHV7 | *Hnrnph1* | Heterogeneous nuclear ribonucleoprotein H RRM domain-containing protein |
| Q6AY09 | *Hnrnph2* | Heterogeneous nuclear ribonucleoprotein H2 |
| P63039 | *Hspd1* | 60 kDa heat shock protein, mitochondrial |
| Q63617 | *Hyou1* | Hypoxia up-regulated protein 1 |
| P56574 | *Idh2* | Isocitrate dehydrogenase [NADP], mitochondrial |
| Q99NA5 | *Idh3a* | Isocitrate dehydrogenase [NAD] subunit alpha, mitochondrial |
| P41565 | *Idh3g* | Isocitrate dehydrogenase [NAD] subunit gamma 1, mitochondrial |
| Q3KR86 | *Immt* | MICOS complex subunit Mic60 |
| P23565 | *Ina* | Alpha-internexin IF rod domain-containing protein |
| Q63416 | *Itih3* | Inter-alpha-trypsin inhibitor heavy chain H3 |
| Q62813 | *Lsamp* | Limbic system-associated membrane protein |
| D3ZHV2 | *Macf1* | Microtubule-actin cross-linking factor 1 |
| O08873 | *Madd* | MAP kinase-activating death domain protein |
| P15205 | *Map1b* | Microtubule-associated protein 1B |
| Q63560 | *Map6* | Microtubule-associated protein 6 |
| Q00566 | *Mecp2* | Methyl-CpG-binding protein 2 |
| Q9JLT0 | *Myh10* | Myosin-10 |
| Q62812 | *Myh9* | Myosin-9 Myosin_tail_1 domain-containing protein |
| Q64122 | *Myl9* | Myosin regulatory light polypeptide 9 |
| Q9QYF3 | *Myo5a* | Unconventional myosin-Va |
| P13596 | *Ncam1* | Neural cell adhesion molecule 1 |
| Q561S0 | *Ndufa10* | NADH dehydrogenase [ubiquinone] 1 alpha subcomplex subunit 10, mitochondrial |
| Q5BK63 | *Ndufa9* | NADH dehydrogenase [ubiquinone] 1 alpha subcomplex subunit 9, mitochondrial |
| Q66HF1 | *Ndufs1* | NADH-ubiquinone oxidoreductase 75 kDa subunit, mitochondrial |
| Q641Y2 | *Ndufs2* | NADH dehydrogenase [ubiquinone] iron-sulfur protein 2, mitochondrial |
| P19234 | *Ndufv2* | NADH dehydrogenase [ubiquinone] flavoprotein 2, mitochondrial |
| P16884 | *Nefh* | Neurofilament heavy polypeptide |
| P19527 | *Nefl* | Neurofilament light polypeptide |
| P12839 | *Nefm* | Neurofilament medium polypeptide |
| Q9QUL6 | *Nsf* | Vesicle-fusing ATPase |
| Q62718 | *Ntm* | Neurotrimin |
| Q2TA68 | *Opa1* | Dynamin-like 120 kDa protein, mitochondrial |
| Q9EPH8 | *Pabpc1* | Polyadenylate-binding protein 1 |
| P52873 | *Pc* | Pyruvate carboxylase, mitochondrial |
| P49432 | *Pdhb* | Pyruvate dehydrogenase E1 component subunit beta, mitochondrial |
| P47858 | *Pfkm* | ATP-dependent 6-phosphofructokinase, muscle type |
| P47860 | *Pfkp* | ATP-dependent 6-phosphofructokinase, platelet type |
| O08662 | *Pi4ka* | Phosphatidylinositol 4-kinase alpha |
| P30427 | *Plec* | Plectin |
| P86252 | *Pura* | Transcriptional activator protein Pur-alpha |
| Q6RUV5 | *Rac1* | Ras-related C3 botulinum toxin substrate 1 |
| Q62703 | *Rcn2* | Reticulocalbin-2 |
| P23358 | *Rpl12* | 60S ribosomal protein L12 |
| P47198 | *Rpl22* | 60S ribosomal protein L22 |
| P61354 | *Rpl27* | 60S ribosomal protein L27 |
| P50878 | *Rpl4* | 60S ribosomal protein L4 |
| P19945 | *Rplp0* | 60S acidic ribosomal protein P0 |
| P62282 | *Rps11* | 40S ribosomal protein S11 |
| P62250 | *Rps16* | 40S ribosomal protein S16 |
| P62703 | *Rps4x* | 40S ribosomal protein S4 40S ribosomal protein S4, X isoform |
| Q6AYT3 | *Rtcb* | RNA-splicing ligase RtcB homolog |
| Q5PPJ9 | *Sh3glb2* | Endophilin-B2 |
| Q63633 | *Slc12a5* | Solute carrier family 12 member 5 |
| P97700 | *Slc25a11* | Mitochondrial 2-oxoglutarate/malate carrier protein |
| P16036 | *Slc25a3* | Phosphate carrier protein, mitochondrial |
| P11167 | *Slc2a1* | Solute carrier family 2, facilitated glucose transporter member 1 |
| P16086 | *Sptan1* | Spectrin alpha chain, non-erythrocytic 1 |
| Q9QWN8 | *Sptbn2* | Spectrin beta chain, non-erythrocytic 2 |
| Q9QXY2 | *Srcin1* | SRC kinase signaling inhibitor 1 |
| Q4FZT0 | *Stoml2* | Stomatin-like protein 2, mitochondrial |
| P61265 | *Stx1b* | Syntaxin-1B |
| P61765 | *Stxbp1* | Syntaxin-binding protein 1 |
| P13086 | *Suclg1* | Succinate--CoA ligase [ADP/GDP-forming] subunit alpha, mitochondrial |
| Q7TP47 | *Syncrip* | Heterogeneous nuclear ribonucleoprotein Q RRM domain-containing protein |
| P70566 | *Tmod2* | Tropomodulin-2 |
| P09495 | *Tpm4* | Tropomyosin alpha-4 chain |
| P68370 | *Tuba1a* | Tubulin alpha-1A chain |
| Q5XIF6 | *Tuba4a* | Tubulin alpha-4A chain |
| Q6AY56 | *Tuba8* | Tubulin alpha-8 chain |
| P85108 | *Tubb2a* | Tubulin beta-2A chain |
| Q3KRE8 | *Tubb2b* | Tubulin beta-2B chain |
| Q4QRB4 | *Tubb3* | Tubulin beta-3 chain |
| Q6P9T8 | *Tubb4b* | Tubulin beta-4B chain |
| P69897 | *Tubb5* | Tubulin beta-5 chain |
| P85834 | *Tufm* | Elongation factor Tu Elongation factor Tu, mitochondrial |
| P32551 | *Uqcrc2* | Cytochrome b-c1 complex subunit 2, mitochondrial |
| Q7TQ16 | *Uqcrq* | Cytochrome b-c1 complex subunit 8 |
| Q9Z2L0 | *Vdac1* | Voltage-dependent anion-selective channel protein 1 |
| P81155 | *Vdac2* | Voltage-dependent anion-selective channel protein 2 |
| Q9R1Z0 | *Vdac3* | Voltage-dependent anion-selective channel protein 3 |
| P31000 | *Vim* | Vimentin |
| Q793F9 | *Vps4a* | Vacuolar protein sorting-associated protein 4A |
| Q9ERH3 | *Wdr7* | WD repeat-containing protein 7 |
| P68255 | *Ywhaq* | 14-3-3 protein theta |


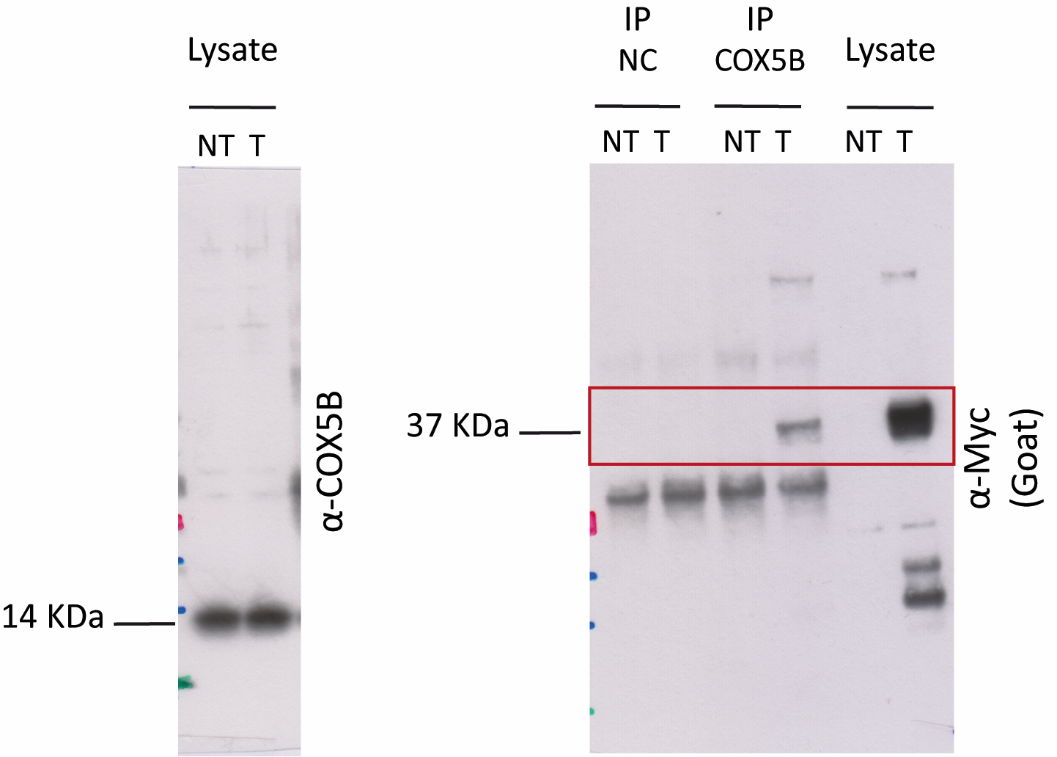


**Supplementary Figure S6**. Co-immunoprecipitation of COX5B and ITM2B using protein extracts from HEK 293 cells transiently transfected with an ITM2B-Myc-tag expression plasmid. The red box highlights the region of ITM2B (37 kDa). Lysate: HEK-293 protein extract; IP: immunoprecipitation with rabbit anti-COX5B and with rabbit anti-HA (ab9110 Abcam) used as negative control (NC).

**
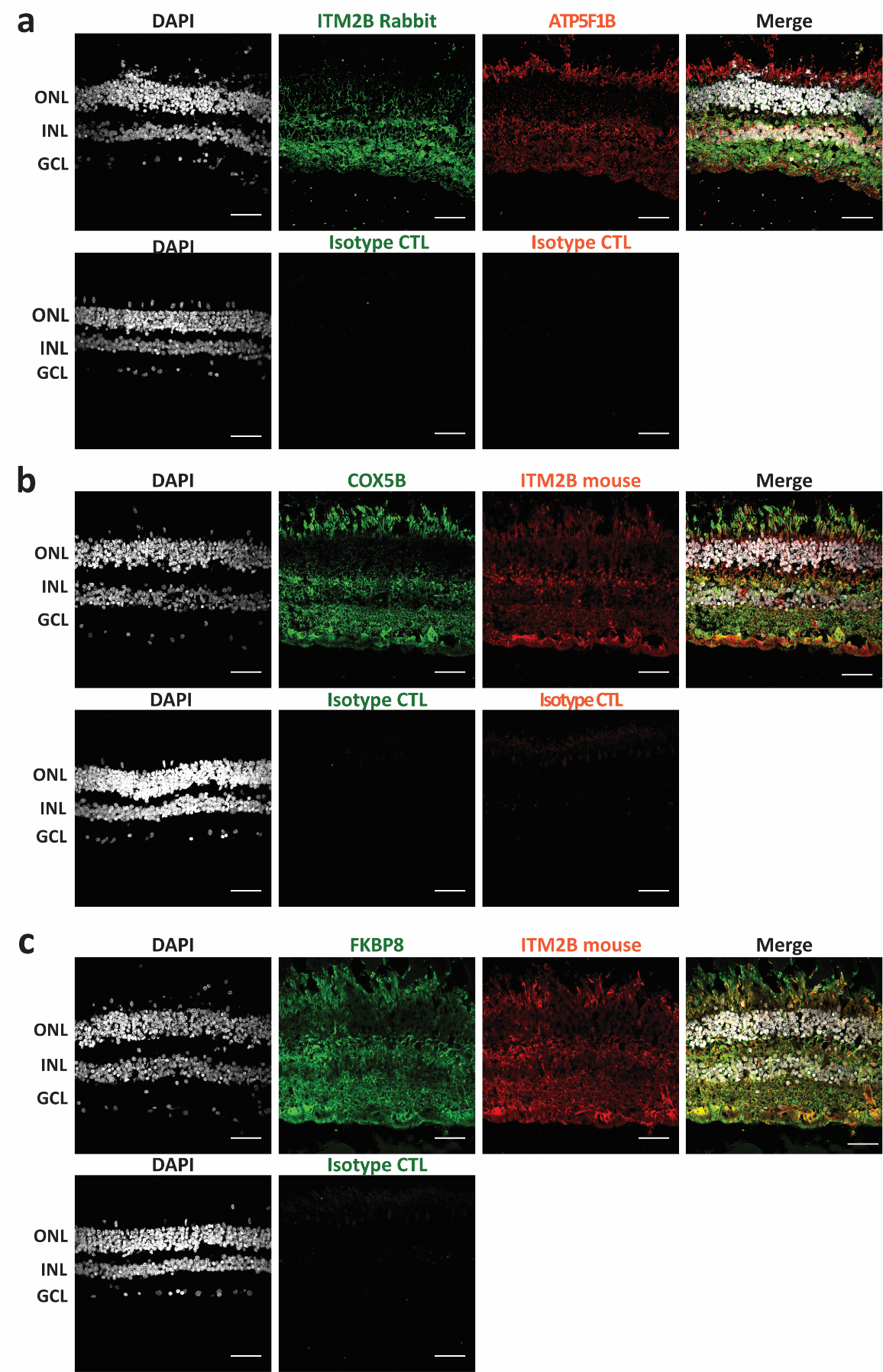
**

**Supplementary Figure S7.** Co-+-localization of ITM2B with the mitochondrial proteins ATP5F1B, COX5B, FKBP8 in the human retina. (a) ITM2B stained with the rabbit antibody (green) partially co-localizes with ATP5F1B (red) in the human retina. (b) ITM2B stained with the mouse antibody (red) co-localizes with COX5B (green) and (c) with FKBP8 (green) in the human retina. ONL: outer nuclear layer; INL: inner nuclear layer; GCL: ganglion cell layer. Scale bar: 50µm. Antibody specificity has been validated using the corresponding isotype controls (CTL).


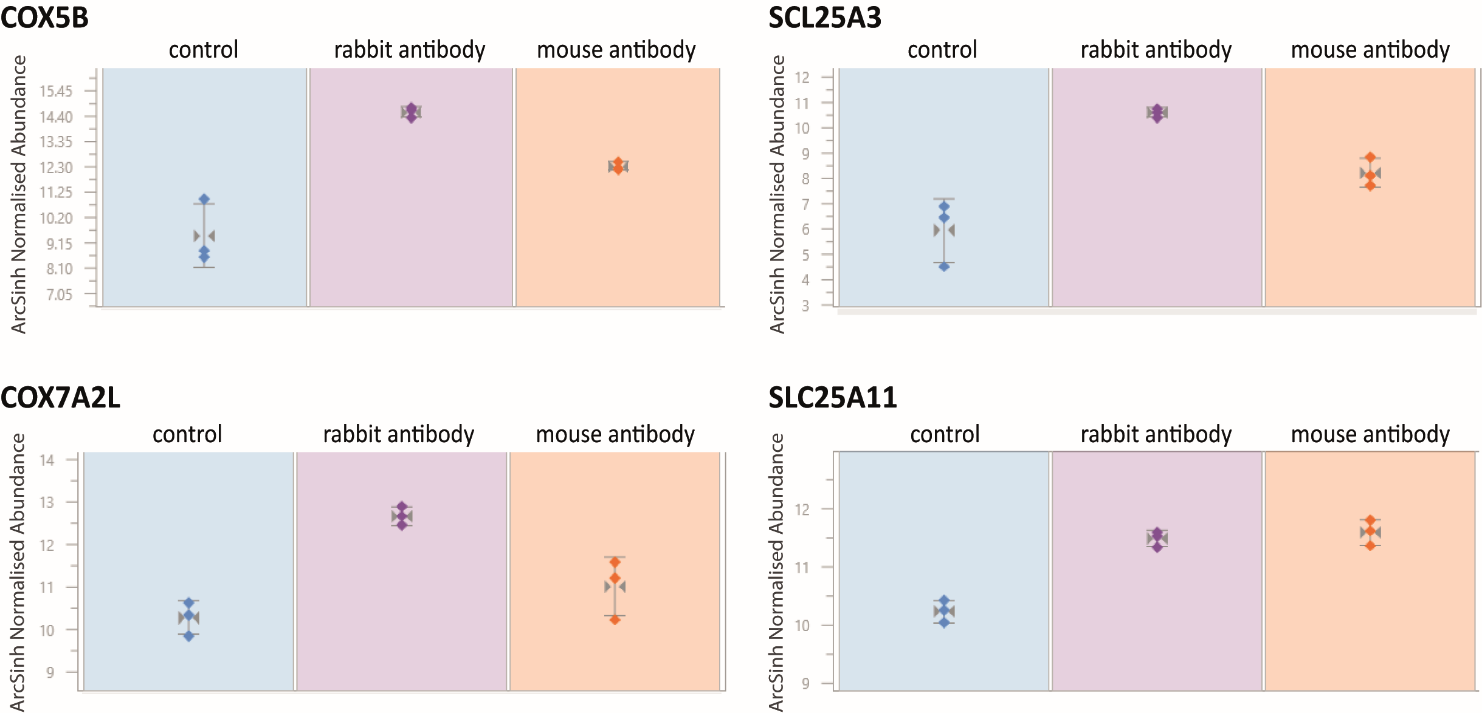


Supplementary Figure S8. Mitochondrial proteins identified with both antibodies in the ITM2B interactome and also present in the ITM2B rat brain interactome. Graphical representation of protein abundance purified with the control unspecific antibody, the rabbit antibody and the mouse antibody using Progenesis QI software.


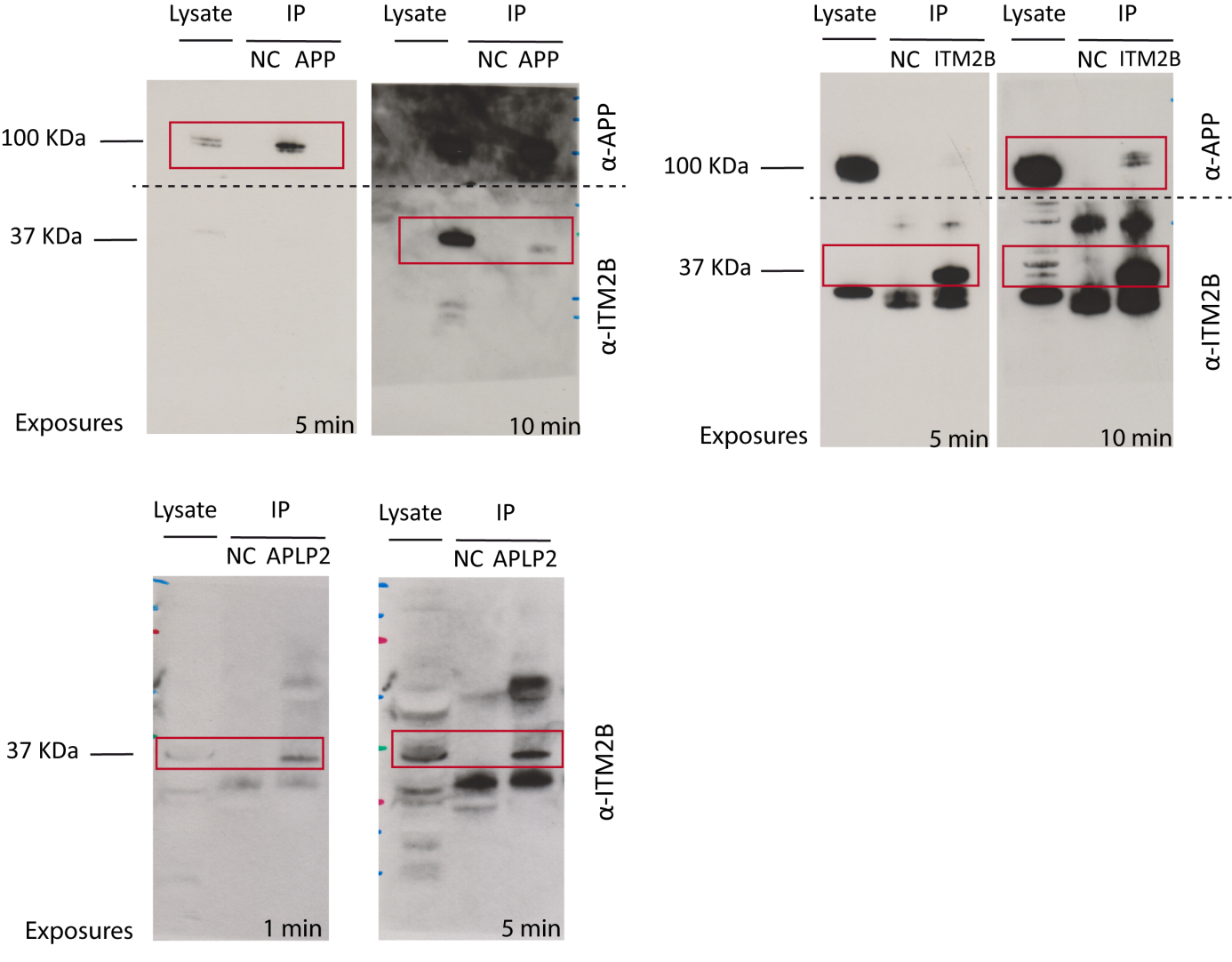


**Supplementary Figure S9.** Original blots referring to Figure 3b. Red boxes highlight the regions of the immunoblots selected for Figure 3b. Lysate: human retinal protein extract; IP: immunoprecipitation; NC: negative control; α-: indicates the antibody used for the staining. Black dotted lines indicate where membranes were cut.


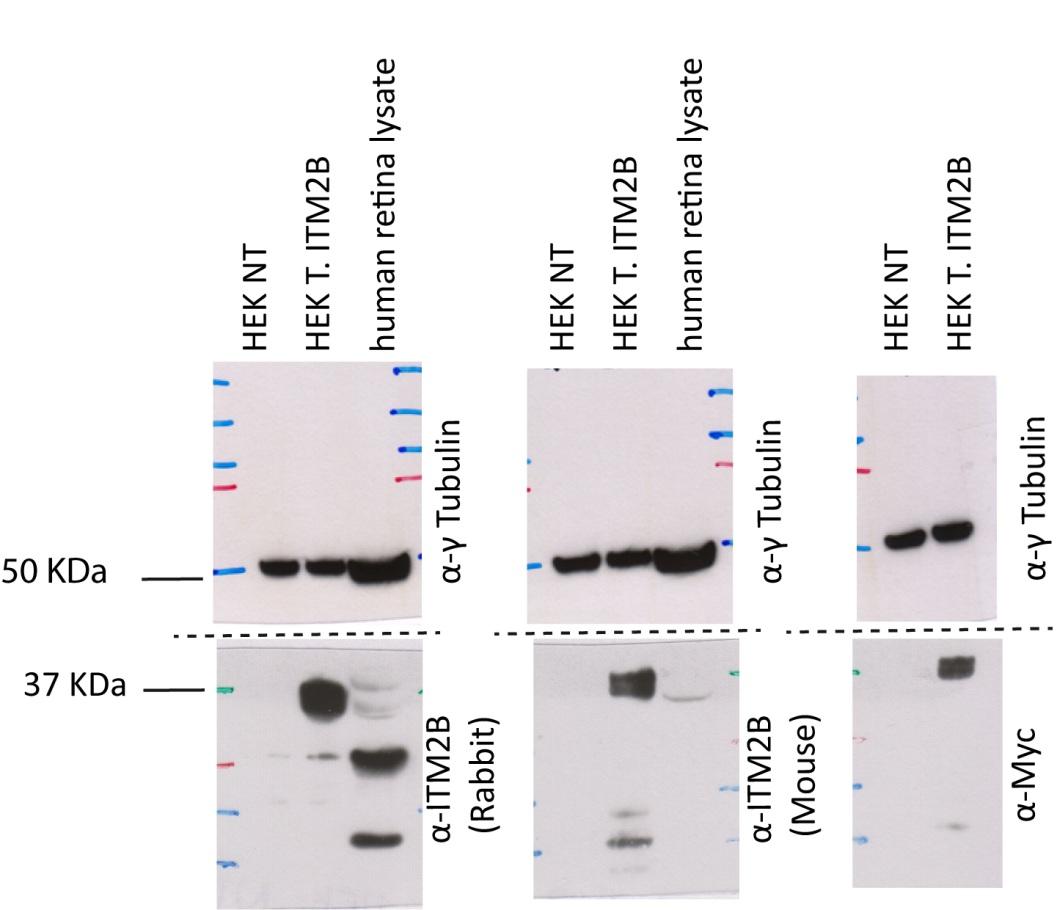


**Supplementary Figure S10.** Original blots referring to Figure 7d. α-: indicates the antibody used for the staining. Black dotted lines indicate where membranes were cut.

# Supplementary Material and Methods

**Supplementary Table S11**: PCR protocol and reaction for *ITM2B* short form

| PCR Mix |  |  | Cycle conditions |  |
| --- | --- | --- | --- | --- |
| Q5 High-Fidelity 2X Master Mix | 12.5 µl |  | 98 °C 1 min |  |
| 1shortF | 1.25 µl |  | 98 °C 10s |  |
| 1shortR | 1.25 µl |  | 63 °C 30s | X40 |
| H_2_0 | 8 µl |  | 72 °C 20s |  |
| Human Retina QUICK-Clone cDNA | 1 µl (2 ng) |  | 72 °C 2 min  10 °C ∞ |  |
| Total | 24 µl |  |  |  |

**Supplementary Table S12**: PCR protocol and reaction for *ITM2B* long form

| PCR Mix |  |  | Cycle conditions |  |
| --- | --- | --- | --- | --- |
| Mix 2 mM | 6.5 µl |  | 95 °C 15 min |  |
| 1longF | 0.5 µl |  | 95 °C 45s |  |
| 1longR | 0.5 µl |  | 60 °C 1 min | X30 |
| H_2_0 | 3 µl |  | 72 °C 1 min |  |
| Human Retina QUICK-Clone cDNA | 2 µl (4 ng) |  | 72 °C 10 min  10 °C ∞ |  |
| Total | 12.5 µl |  |  |  |

***HEK transfection/Immunoprecipitation / Interaction validation by immunoblotting***

HEK transfection, protein extraction, immunoprecipitation and immunoblotting were performed as described in the Material and Methods’ section. Twenty µl of Dynabead protein G (Thermo Fisher Scientific, Waltham, USA) were used to perform immunoprecipitation with 50 µg of protein lysate. The antibodies used for these experiments are listed in the supplementary Table S13.

**Supplementary Table S13**: List of primary and secondary antibodies used for immunoprecipitation and Western blots

| **Antigen** | **Species** | **Dilution** | **Source** |
| --- | --- | --- | --- |
| ITM2B | Mouse  Rabbit | IP: 2 µg; WB: 1/500  IP: 2 µg; WB: 1/500 | Santa Cruz (sc-374362)  Thermo Fisher Scientific (PA5-31441) |
| COX5B | Rabbit | IP: 2 µg; WB: 1/500 | Thermo Fisher Scientific (PA5-96189) |
| Myc | Mouse  Rabbit  Goat | IP: 2 µg; WB: 1/500  IP: 2 µg; WB: 1/500  IP: 2 µg; WB: 1/1000 | Millipore (116671490001)  Abcam (ab9106)  Abcam (ab9132) |
| anti-HA | Rabbit | IP: 2 µg; | Abcam (ab9110) |
| Mouse HRP | Donkey | WB: 1/20 000 | Jackson ImmunoResearch (715-035-150) |
| Rabbit HRP | Donkey | WB: 1/20 000 | Jackson ImmunoResearch (111-035-003) |

*IP: immuno-precipitation, WB: Western blot, HR: horseradish peroxidase*

***Preparation of retinal sections and Immunofluorescence***

A human retina was fixed in 4% PFA, embedded in 7, 5% gelatin-10% sucrose and the block was frozen in isopentan at - 45°C. Fourteen-µm sections were obtained using a Cryostat (NX70, Microm Microtech France). Different post fixation steps were carried out on slides before immunostaining. Cold methanol (rabbit ITM2B and ATP5F1B antibodies) and ethanol (mouse ITM2B, COX5B and FKBP8 antibodies) were applied for 5 minutes. After washing in PBS 1X, sections were treated with PBS 1X, 0.2 % gelatin, 0.25% Triton-X100 (blocking solution) for 1h at room temperature (RT). Primary antibodies diluted in blocking solution were added to the slides overnight at 4°C. Slides were washed three times with PBS 1X, 0.1% Triton-X100 before adding the secondary antibodies and DAPI diluted in blocking buffer for 1.5 hours at RT. An inverted confocal microscope (Olympus, Tokyo, Japan) was used to image the fluorescent staining. To assess the specificity of primary antibodies, isotypic controls were used. Primary antibodies, isotypic controls and secondary antibodies are listed below.

**Supplementary Table S14**: List of primary and secondary antibodies used for immunofluorescence.

| **Antigen** | **Species** | **Dilution** | **Source** |
| --- | --- | --- | --- |
| ITM2B | Mouse  Rabbit | 1/250  1/500 | Santa Cruz (sc-374362)  Thermo Fisher Scientific (PA5-31441) |
| ATP synthase beta  (ATP5F1B) | Mouse | 1/1000 | Thermo Fisher Scientific (A-21351) |
| COX5B | Rabbit | 1/100 | Thermo Fisher Scientific (PA5-96189) |
| FKBP8 | Rabbit | 1/500 | Sigma-Aldrich (ABS992) |
| IgG  Isotype  Control | Mouse | According to the specific antibody used | Cell Signaling (#5415) |
| IgG  Isotype  Control | Rabbit | According to the specific antibody used | Cell Signaling (#3900) |
| Alexa Fluor-488 anti-rabbit | Donkey | 1/1000 | Jackson ImmunoResearch (711-545-152) |
| Cy3 anti-mouse | Donkey | 1/1000 | Jackson ImmunoResearch (715-165-150) |

# Supplementary Reference

1 Martins, F. *et al.* Identification and characterization of the BRI2 interactome in the brain. *Sci Rep* **8**, 3548, doi:10.1038/s41598-018-21453-3 (2018).
